# Remdesivir-related cost-effectiveness and cost and resource use evidence in COVID-19: a systematic review

**DOI:** 10.1007/s15010-022-01930-8

**Published:** 2022-10-12

**Authors:** Molly Murton, Emma Drane, James Jarrett, Oliver A. Cornely, Alex Soriano

**Affiliations:** 1 Costello Medical, London, UK; 2 Costello Medical, Cambridge, UK; 3 Gilead Sciences, Inc., London, UK; 4 Faculty of Medicine and University Hospital Cologne, Department I of Internal Medicine, Excellence Centre for Medical Mycology (ECMM), University of Cologne, Cologne, Germany; 5 Faculty of Medicine and University Hospital Cologne, Cologne Excellence Cluster On Cellular Stress Responses in Aging-Associated Diseases (CECAD), University of Cologne, Cologne, Germany; 6 Faculty of Medicine and University Hospital Cologne, Clinical Trials Centre Cologne (ZKS Köln), University of Cologne, Cologne, Germany; 7 German Centre for Infection Research (DZIF), Partner Site Bonn-Cologne, Cologne, Germany; 8 Department of Infectious Diseases, Hospital Clínic of Barcelona, University of Barcelona, IDIBAPS, Barcelona, Spain

**Keywords:** Remdesivir, COVID-19, SARS-CoV-2, Economic burden

## Abstract

**Background:**

The coronavirus disease 2019 (COVID-19) pandemic has been a global health emergency since December 2019, leading to millions of deaths worldwide and placing significant pressures, including economic burden, on individual patients and healthcare systems. As of February 2022, remdesivir is the only US Food and Drug Administration (FDA)-approved treatment for severe COVID-19. This systematic literature review (SLR) aimed to summarise economic evaluations, and cost and resource use (CRU) evidence related to remdesivir during the COVID-19 pandemic.

**Methods:**

Searches of MEDLINE, Embase the International Health Technology Assessment (HTA) database, reference lists, congresses and grey literature were performed in May 2021. Articles were reviewed for relevance against pre-specified criteria by two independent reviewers and study quality was assessed using published checklists.

**Results:**

Eight studies reported resource use and five reported costs related to remdesivir. Over time, the prescription rate of remdesivir increased and time from disease onset to remdesivir initiation decreased. Remdesivir was associated with a 6% to 21.3% decrease in bed occupancy. Cost estimates for remdesivir ranged widely, from $10 to $780 for a 10-day course. In three out of four included economic evaluations, remdesivir treatment scenarios were cost-effective, ranging from ~ 8 to ~ 23% of the willingness-to-pay threshold for the respective country.

**Conclusions:**

Economic evidence relating to remdesivir should be interpreted with consideration of the broader clinical context, including patients’ characteristics and the timing of its administration. Nonetheless, remdesivir remains an important option for physicians in aiming to provide optimal care and relieve pressure on healthcare systems through shifting phases of the pandemic.

**Supplementary Information:**

The online version contains supplementary material available at 10.1007/s15010-022-01930-8.

## Introduction

Since the beginning of the COVID-19 pandemic in December 2019, more than 418 million cases have been reported worldwide across 223 countries and territories, with more than 5.8 million deaths [1–3]. COVID-19 is caused by the novel severe acute respiratory syndrome coronavirus 2 (SARS-CoV-2) and is highly transmissible [4].

The clinical course and symptoms of COVID-19 can range from asymptomatic to resembling a common cold to severe life-threatening conditions that require hospitalisation [5]. Patients with progressively worsening disease may require extended intensive care unit (ICU) stays, invasive mechanical ventilation (IMV) and extracorporeal membrane oxygenation (ECMO). Based on various cohort studies, approximately 19% of patients hospitalised with COVID-19 are admitted to the ICU, and 16% receive IMV [6, 7]. Hospitalisation, in particular ICU stay and IMV, are associated with high clinical burden for patients; for example including increased risk for coma, infection, ventilator-related injuries, and pneumonia [8, 9]. In addition, there is a substantial burden on health systems. Surges in hospitalisations have overwhelmed hospital capacities, severely undermining their ability to deliver care to patients with COVID-19, alongside those with other conditions [10, 11]. While vaccines may lessen the healthcare burden, in many parts of the world, there are still significant barriers to vaccine uptake [12–14]. Therefore, there remains a need for effective treatments that can improve patient outcomes and relieve the pressure on healthcare systems.

Recently, emergency use authorisation from the US FDA has been granted for two novel antivirals, molnupiravir and paxlovid, as well as the existing antiviral remdesivir, for the treatment of non-hospitalised patients with mild-to-moderate illness [15–17]. However, as of February 2022, remdesivir is the only FDA-approved treatment for severe COVID-19, indicated for COVID-19 requiring hospitalisation in patients aged 12 and above. It also has conditional marketing authorisation from the European Medicines Agency [18].

Several large clinical trials have reported results demonstrating remdesivir’s potential to reduce burden on patients and health systems; for example, shorter time to recovery, higher odds of clinical improvement and reduced need for mechanical ventilation in patients receiving remdesivir compared with placebo [19–21].

This SLR aimed to identify and critically appraise the economic evidence on remdesivir to provide an overview that could inform future HTAs and assessment of the impact of further adoption of remdesivir in clinical practice.

## Methods

An SLR was conducted in July 2020 and updated in May 2021 in accordance with a pre-specified protocol and the methodological principles of conduct for SLRs as detailed in the University of York Centre for Reviews and Dissemination’s guidance [22].

### Identification of evidence

A comprehensive approach was taken to evidence identification. Electronic database searches were conducted in MEDLINE, Embase and the International HTA database. Manual hand searches of key conference proceedings from the last 2 years; HTA body websites and health economics databases; press releases from relevant manufacturer and trial websites and bibliographies of any relevant SLRs, (network) meta-analyses or HTAs identified during the review were performed. In addition, given potential poor indexing of publications relating to COVID-19 in electronic databases [23], supplementary targeted searches of Google and pre-print sources (MedRxiv and Social Science Research Network) were performed. Complete search strategies are presented in the Supplementary Appendix.

### Selection of studies and data extraction

Pre-defined eligibility criteria for article inclusion were developed using the Population, Intervention, Comparison and Outcomes framework (Supplementary Appendix). Studies were required to be original peer-reviewed research, HTAs or recent congress abstracts that included unselected patients aged 12 or older with COVID-19 and investigated remdesivir, either alone or in combination with other therapies. Relevant outcomes included cost-effectiveness results, such as incremental cost-effectiveness ratios or quality-adjusted life-years (QALYs); healthcare system costs; societal costs and resource use.

Studies were reviewed against the eligibility criteria by two independent reviewers in two stages. First, titles and abstracts were screened for the removal of clearly irrelevant articles, followed by the full texts. Review of articles from the supplementary searches was conducted by a single reviewer with input provided by a second individual in cases of uncertainty. All included records were confirmed by a second reviewer.

Key information from included studies was extracted into pre-specified data extraction tables by a single individual, and independently verified by a second individual.

### Quality assessment

The quality of all included economic evaluations was assessed using the Drummond checklist [24]. The quality of all included CRU studies was assessed using the Alberta Heritage Foundation for Medical Research (AHFMR) checklist for quantitative studies [25]. Quality assessments were completed by one individual and verified by a second individual.

## Results

**Fig. 1:**
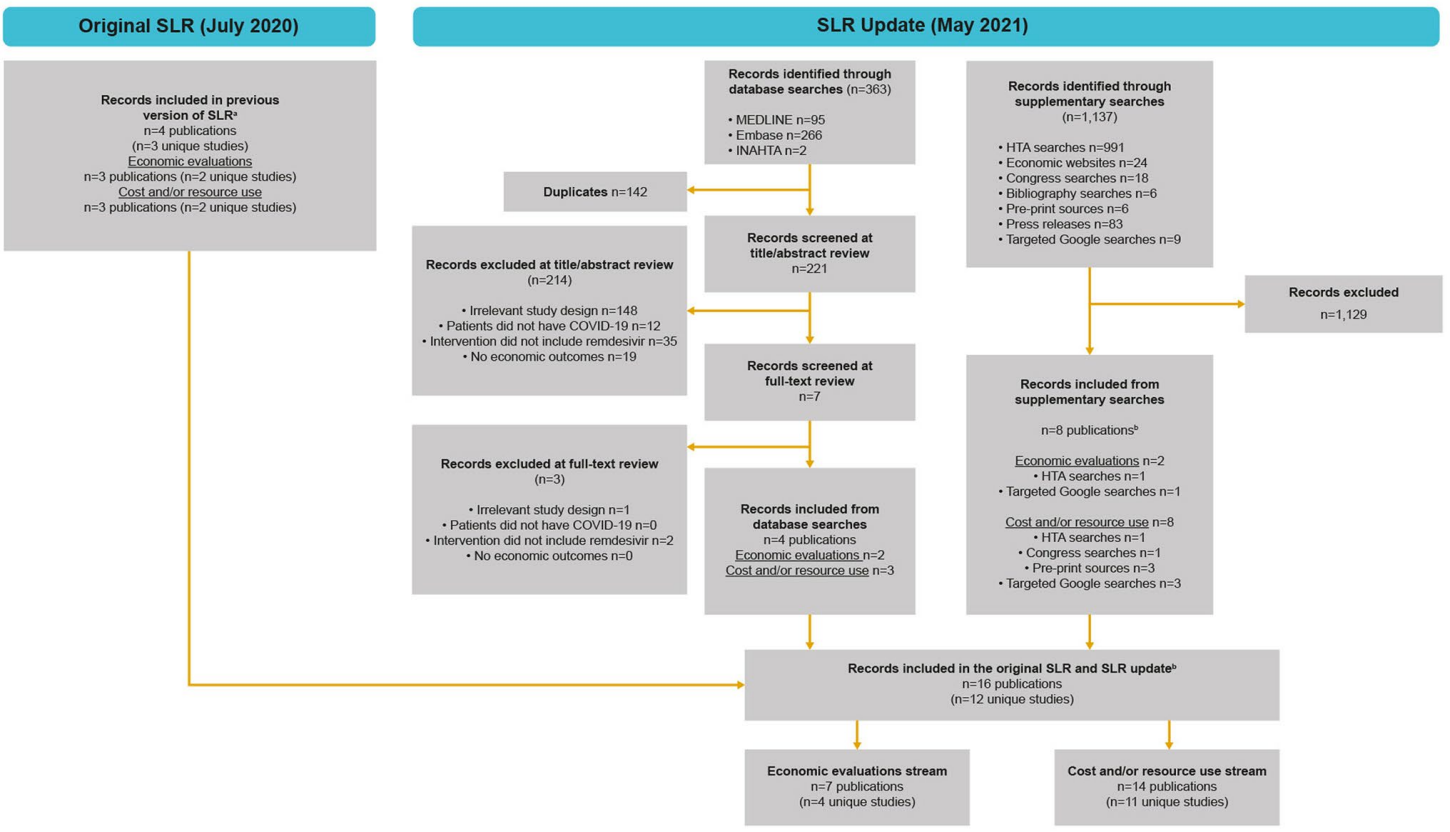
PRISMA flow diagram. ^a^One included study was an economic evaluation only, one only reported cost and resource use outcomes, and one reported economic evaluation, utilities and cost and resource use. ^b^Two studies were included in all three streams of the SLR; one study was included in economic evaluations and cost and resource use. *HTA* Health Technology Assessment, *PRISMA* Preferred Reporting Items for Systematic Reviews and Meta-Analyses, *SLR* systematic literature review

The number of included and excluded studies is presented in a Preferred Reporting Items for Systematic Reviews and Meta-Analyses (PRISMA) flow diagram (Fig. 1) [26]. After the removal of duplicates and irrelevant articles, 11 CRU studies and 4 economic evaluations were ultimately included.

### Cost and resource use studies

#### Study characteristics

Characteristics of the identified CRU studies are summarised in Table 1. Most were conducted in the US (*n* = 4) [27–30], followed by Spain (*n* = 2) [31, 32]. One each was conducted in the UK [33], France [34], China [35], Bangladesh [36] and internationally [37]. Currency and/or currency year was reported in five studies. Of these, three studies used 2020 US Dollars [28, 30, 37], one study used Euros (cost year not reported) [34], and one study used 2020 Chinese Yuan (CN¥) [35] in cost calculations. Most studies either used a healthcare system perspective (*n* = 4) [28, 32, 34, 35], or an individual hospital perspective (*n* = 3) [27, 31, 33].

**Table 1:** Characteristics of included CRU studies

Study name	Country	Perspective	Currency year	Sample size	Population characteristics	Clinical and cost data source(s)
Anderson 2021	US	Emergency departments at two US hospitals	NR	1643	≥ 18 years with severe hospitalised COVID-19	NR
Bechman 2021	UK	London hospital	NR	No remdesivir (*n* = 1469); remdesivir (*n* = 737)	≥ 16 years admitted as an emergency with COVID-19 between 1st March 2020 and 25th January 2021	NR
Béraud 2021	France	French healthcare system	Currency: EUR, year: NR	NR	NR (results reported in two sub-populations: only low-flow patients and low-flow + high-flow patients)	Previously published (Hoertel 2020) stochastic agent-based model calibrated to the French setting combined with French epidemiological data on COVID-19 and data from ACTT-1 (remdesivir data) and RECOVERY trial (dexamethasone data) Costs were sourced from the DRG-based national hospital tariffs to estimate the daily cost of inpatient care in conventional and ICU wards Additional costs for COVID-19 care were calculated using the additional expenses received by hospitals (Sabine 2020) and the number of hospitalised patients (GEODES 2020)
Garcia-Vidal 2021	Spain	Spanish hospital perspective	NR	1645	All consecutive patients admitted ≥ 48 h to Hospital Clinic of Barcelona for COVID-19 between 1st March 2020 and 30th September 2020; 88.4% Caucasian	Electronic health records
Hill 2020	International	NR	2020 USD	NR	NR	The minimum costs of drug production were estimated by calculating the cost of API, combined with costs of excipients, formulation, packaging and a profit margin Remdesivir API production costs were estimated based on published second-generation routes of chemical synthesis and assumed overheads such as occupancy rate per hour and labour (National Center for Biotechnology Information, Siegal 2017)
ICER 2020	US	Healthcare system	2020 USD	NR	NR	The price estimate of remdesivir includes: the marginal cost of producing the next course of remdesivir therapy; research and development costs provided by the manufacturer; and research and development costs provided by the federal government Minimum cost of production for remdesivir was sourced from Hill 2020 Sale price of remdesivir was estimated to be $600 after studying the prices announced by Beximco, Hetero and Cipla Federal investment in the earlier phases of research was sourced from Knowledge Ecology International Costs spent by remdesivir sponsor (Gilead) was sourced from public statements made by Gilead (approximately $1 billion) Costs for hospitalisation were calculated by dividing total visit costs (sourced from Rae 2020) by length of time in hospital (sourced from ACTT-1)
Jiang 2021	China	Healthcare system	2020 CN¥	NR	Patients hospitalised with COVID-19	One-time visit costs based on assumption
Mozaffari 2021	US	Real-world utilisation	NR	190,529	Adult patients admitted between May 1st and November 30th 2020 with a primary or secondary discharge diagnosis	Premier Healthcare Database
Nasir 2021	Bangladesh	NR	NR	99	Patients admitted to the ICU with COVID-19 between May and September 2020	Non-electronic hospital records and treatment sheets
Sheinson 2021	US	Health payer and societal	2020 USD	NR	NR	Bundled payments were estimated by a weighted average of costs to commercial payers, Medicare and Medicaid Medicare payments were taken from reimbursement rates released by Centers for Medicare and Medicaid Services, Medicaid payments were assumed equal to those for Medicare, and commercial payments were estimated using 2017 cost data (adjusted to 2020 USD) from the Healthcare Cost and Utilization Project DRGs were used to calculate the bundled payments costs for each level of oxygen support In the absence of data on the exact services rendered for different levels of care for COVID-19 hospitalisations, the FFS per diem costs were derived by dividing average bundled payments by average LOS for the corresponding DRGs from the HCUP data (for commercial patients) or from published phase 3 trials (ACTT) Additional healthcare costs for patients discharged after receiving mechanical ventilation inpatient were applied in the first-year post-discharge based on Ruhl et al. Productivity costs by age were sourced from Grosse 2009
Soriano 2021	Spain	Spanish national	NR	NR	Patients hospitalised with COVID-19 and pneumonia requiring low-flow oxygen therapy at the time of hospitalisation from January 31st 2020 to May 10th 2020 (patients who are eligible for remdesivir based on SNHS guidance)	Population data were sourced from Instituto de Salud Carlos III and the published literature (Gold 2020) Resource use was sourced from published data using a literature review, with international references used whenever national data were not available (Beigel 2020, Instituto de Salud Carlos III, Casas 2020, and Corregidor-Luna 2020) The number of beds in a general ward and ICUs were sourced from the Ministry of Health

*ACTT-1* adaptive COVID-19 treatment trial, *API* active pharmaceutical ingredients, *CRU* cost and resource use, *CN¥* Chinese Yuan, *DRG* diagnosis-related group, *EUR* Euro, *FFS* fee-for-service, *ICU* intensive care unit, *NR* not reported, *SNHS* Spanish National Health Service, *USD* United States Dollars

#### Study results

**Fig. 2:**
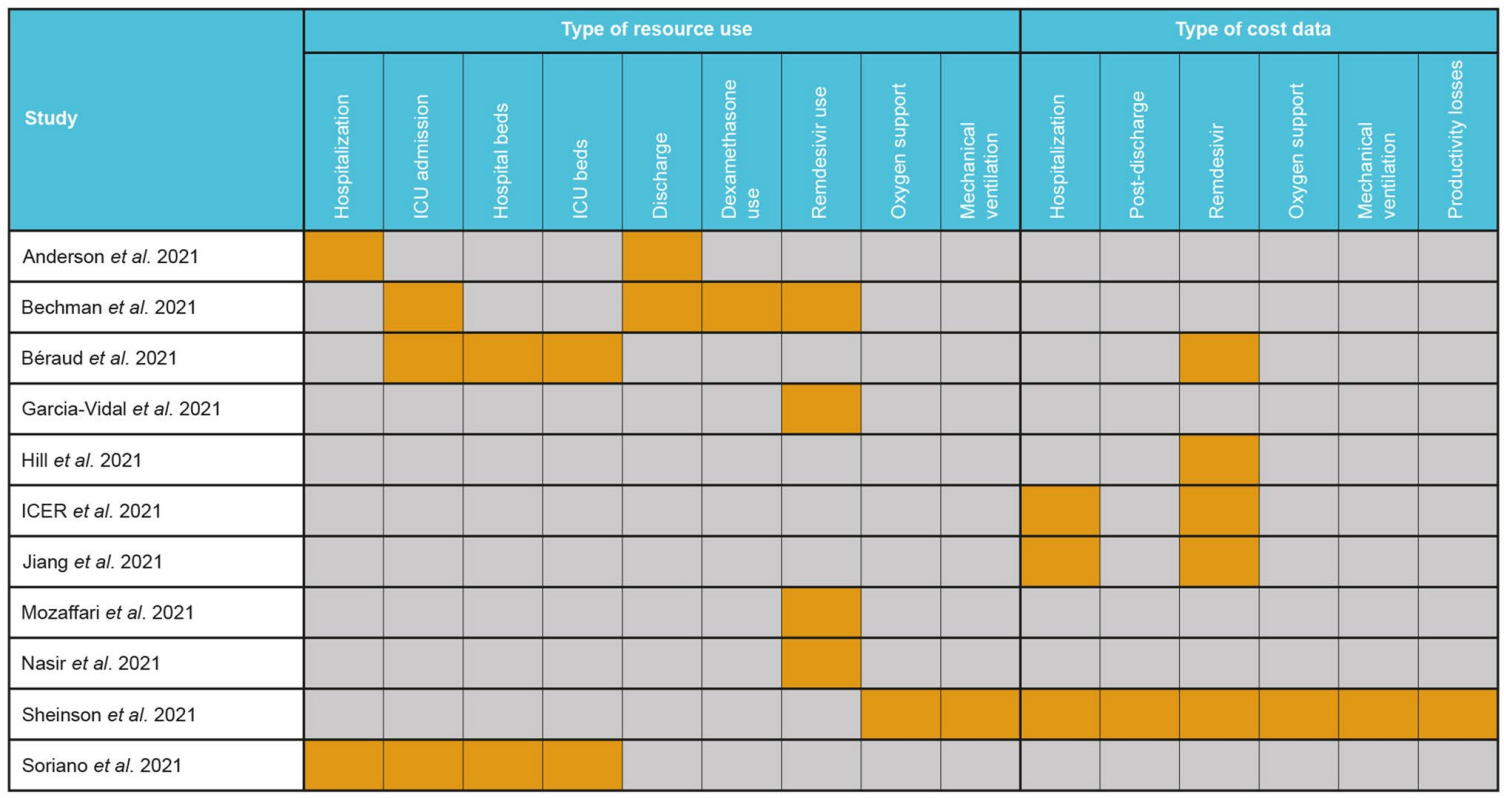
Summary of studies reporting CRU data. *CRU* cost and resource use, *ICU* intensive care unit

**Fig. 3:**
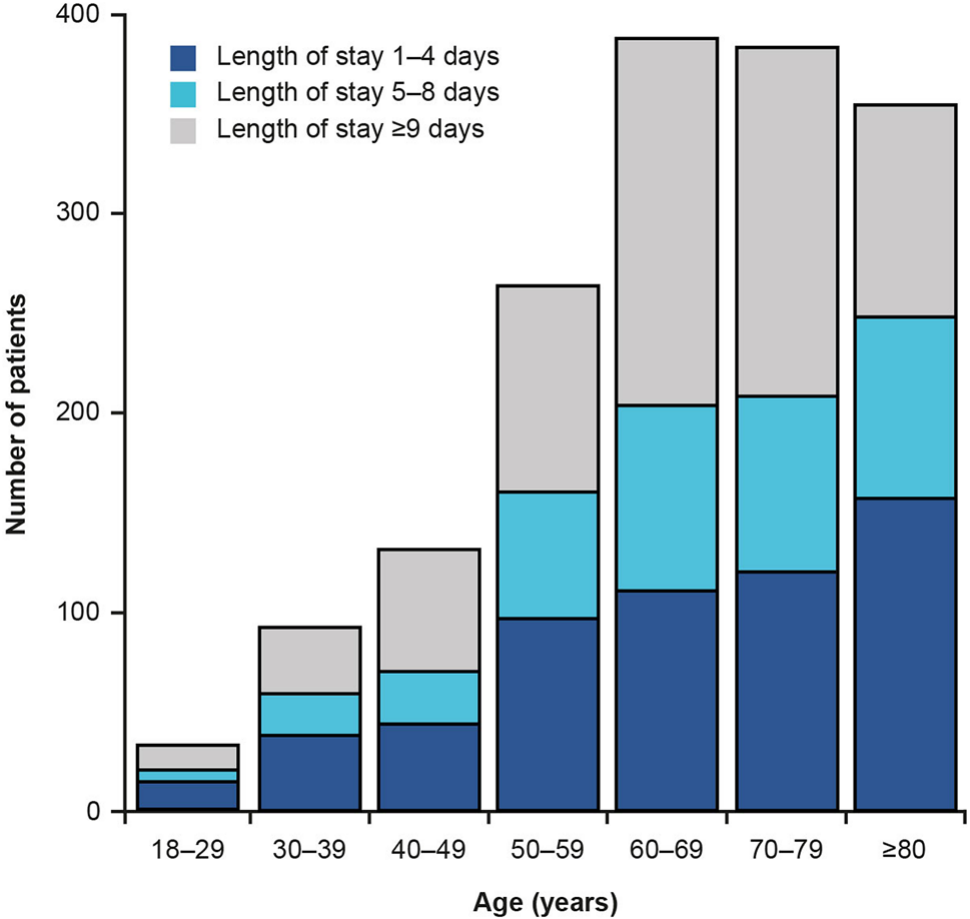
Frequency histogram by age groups for hospital LOS reported in Anderson 2021. Adapted from Anderson 2021 with permission. *LOS* length of stay

Eight studies reported resource use related to remdesivir (Fig. 2). In a cohort of 1643 adults treated with remdesivir in a US hospital, length of stay (LOS) was 1–4 days in 36%, 5–8 days in 23% and ≥ 9 days in 41% of patients [27]. The proportion of patients in each LOS category was consistent across all age groups (Fig. 3). In addition, patients with shorter LOS were more frequently discharged to home (~ 60% in those with 1–4 or 5–8 days, versus 33% for ≥ 9 days) and had lower rates of admission to rehabilitation centres (6%, 12%, and 28%, respectively) [27].

**Fig. 4:**
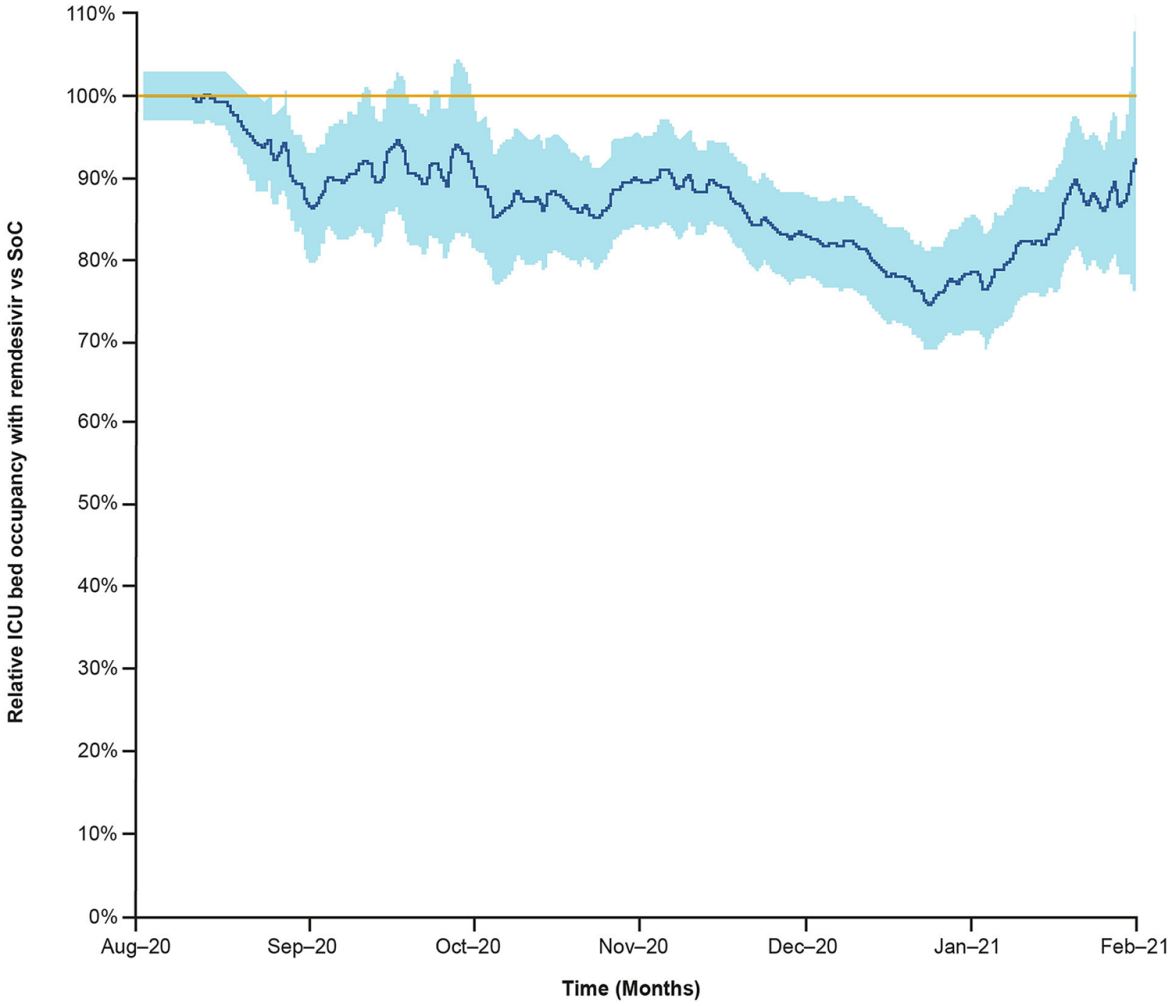
ICU bed occupancy gains associated with remdesivir compared with SoC if all eligible low- and high-flow oxygen patients are treated. Adapted from Béraud 2021. *ICU* intensive care unit, *SoC* standard of care

Two epidemiological modelling studies evaluated hospital and ICU bed occupancy, both using data from the adaptive COVID-19 treatment (ACTT-1) trial supplemented by regional epidemiology and cost data [32, 34]. Béraud 2021 found that administering remdesivir between August 2020 and February 2021 would have resulted in lower bed occupancy in France (Fig. 4), with a daily gain of 321.3 beds if all eligible low- and high-flow oxygen patients were treated. During the peak of the second wave of the pandemic in France (18 November 2021), bed occupancy would have been 16% lower in the ICU if all eligible low- and high-flow oxygen patients were treated [34]. Similarly, Soriano 2021 reported that remdesivir would have prevented at least half of the ICU admissions during the first wave of the pandemic in Spain. During maximum occupancy throughout the first wave, remdesivir would have resulted in a 17.53% and a 23.98% increase in available beds in general wards and in the ICU, respectively [32].

**Fig. 5:**
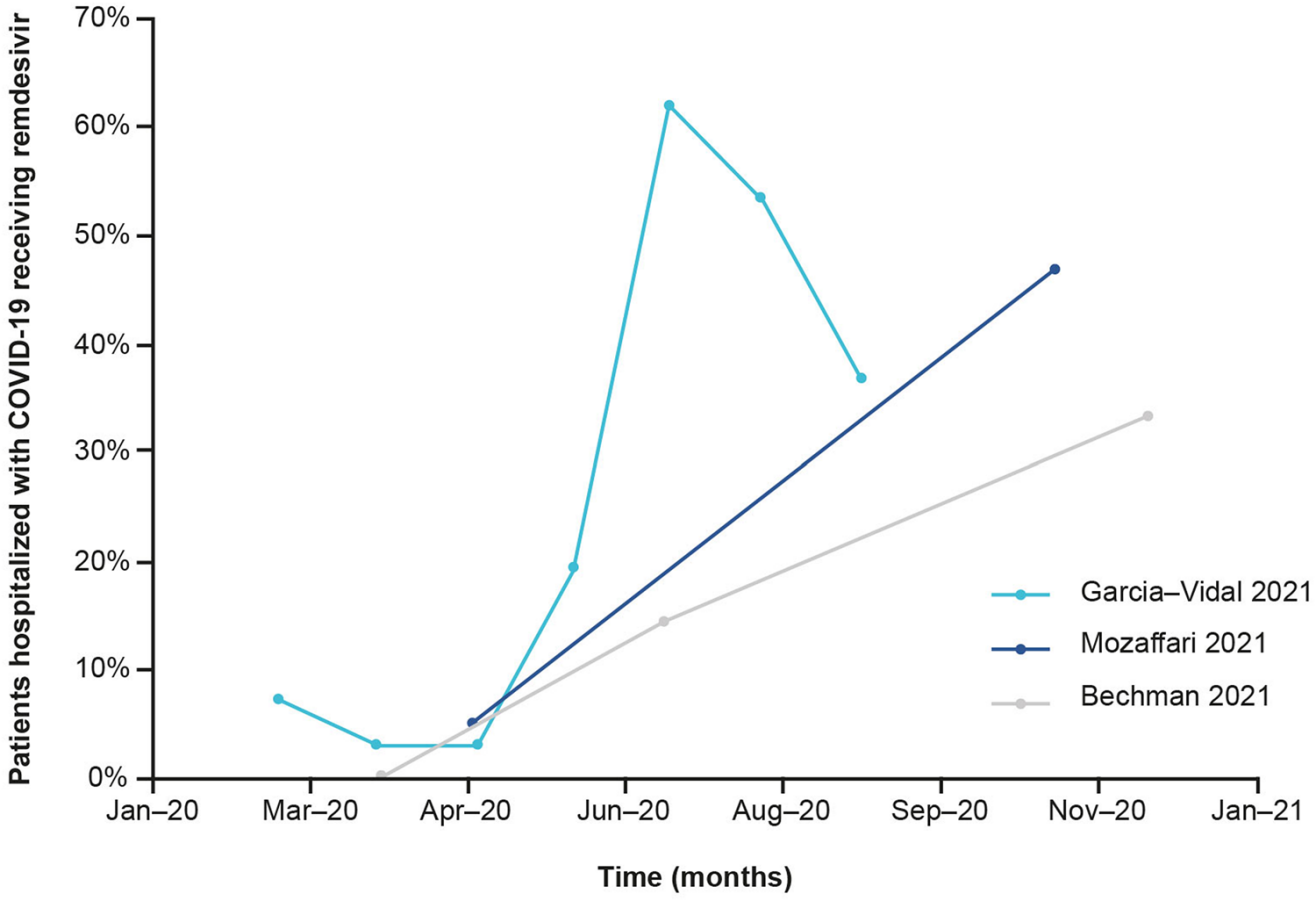
Prescription of remdesivir over time. Bechman 2021 reported prescription frequencies for three-time frames: wave 1, wave 2 and the period between waves. Wave 1 was defined as 5th March 2020 to 7th May 2020; wave 2, defined as 18th October 2020 to ongoing at the time of analysis on 25th January 2021. Data were plotted at the approximate midpoint of each time period

In three studies assessing the frequency of remdesivir prescription between March and November 2020 (towards the beginning of the pandemic), a general increase was seen (**Fig. ****5**) [29, 31, 33]. Bechman 2021, a UK-based observational study including 3,595 patients, reported that remdesivir was prescribed to 0.1% of hospitalised patients during the first wave of the pandemic, 14.5% during the period between the first and second wave and 33.4% in the second wave [33]. Meanwhile, a Spanish hospital cohort study of 1,645 patients, Garcia-Vidal 2021, reported that remdesivir use increased from 3% in April 2020 to 62% in July 2020 in hospitalised patients, but decreased in August and September 2020, to 53% and 36.5% [31]. In both studies, similar trends were observed for the prescription of dexamethasone, suggesting that remdesivir and dexamethasone were used together. Indeed, Bechman 2021 also reported that 98.8% of patients receiving remdesivir were co-prescribed dexamethasone [33]. Mozaffari 2021, a US-based real-world utilisation study also found that the percentage of remdesivir use increased, from 5% in May 2020 to 47% in November 2020 [29]. Both the Spanish and US study also reported time from symptom onset to remdesivir prescription. Garcia-Vidal 2021 reported a reduction from 15 days in June to 6 days in August 2020 [31], while Mozaffari 2021 reported that the percentage of patients receiving remdesivir within the first two days of hospitalisation increased from 40% in May to 85% in November 2020 [29].

Five studies assessed the cost associated with remdesivir and related resource use (Fig. 2) [28, 30, 34, 35, 37]. Béraud 2021 reported that the bed occupancy gains associated with remdesivir treatment for an average of 4 days would result in savings ranging from €307.30 if all eligible low-flow oxygen patients were treated to €720.63 if all eligible low- and high-flow oxygen patients were treated. In the reported worst-case scenario (only eligible low-flow oxygen patients treated for 5 full days), results would be neutral with an added cost of €37.70 [34].

Hill 2020 aimed to estimate the cost of manufacturing generic remdesivir based on the individual costs of active pharmaceutical ingredients, combined with costs of excipients, formulation, packaging, and a profit margin. Using this calculation, the price of a 10-day course of treatment was found to be $9.27, equating to $0.93 per day [37]. The 2020 HTA report from the Institute for Clinical and Economic Review (ICER), used this result from Hill 2020 to estimate the lower bound cost of a 10-day course of remdesivir at $10 and a 5-day course at $5 [28]. ICER estimated the higher bound cost of a 10-day course at $600, based on manufacturers’ reports from Bangladesh and India. Based on this, ICER estimated a cost recovery price for remdesivir, which it defined as a pricing paradigm that assumes the only goal is to set a price that compensates the manufacturer for the costs of production without additional profit [38] and reported that this would range from $5 to $600 if accounting for the minimal marginal cost only. In addition, accounting for the manufacturer’s projected 2020 research and development costs, the cost recovery price would range from $1005 to $1600 [28].

Sheinson 2021 evaluated costs associated with a treatment regimen of remdesivir and dexamethasone across bundled (a model in which all aspects of a patient’s care for a given condition are bundled into one price) and fee-for-service (FFS; a model in which each aspect of care is paid for separately) payment models, including post-discharge costs. Short-term hospital costs were estimated as $22,385 and $22,076 for bundled and FFS payments, respectively. In addition, productivity losses associated with COVID-19 amounted to $18,279 [30].

#### Study quality

**Fig. 6:**
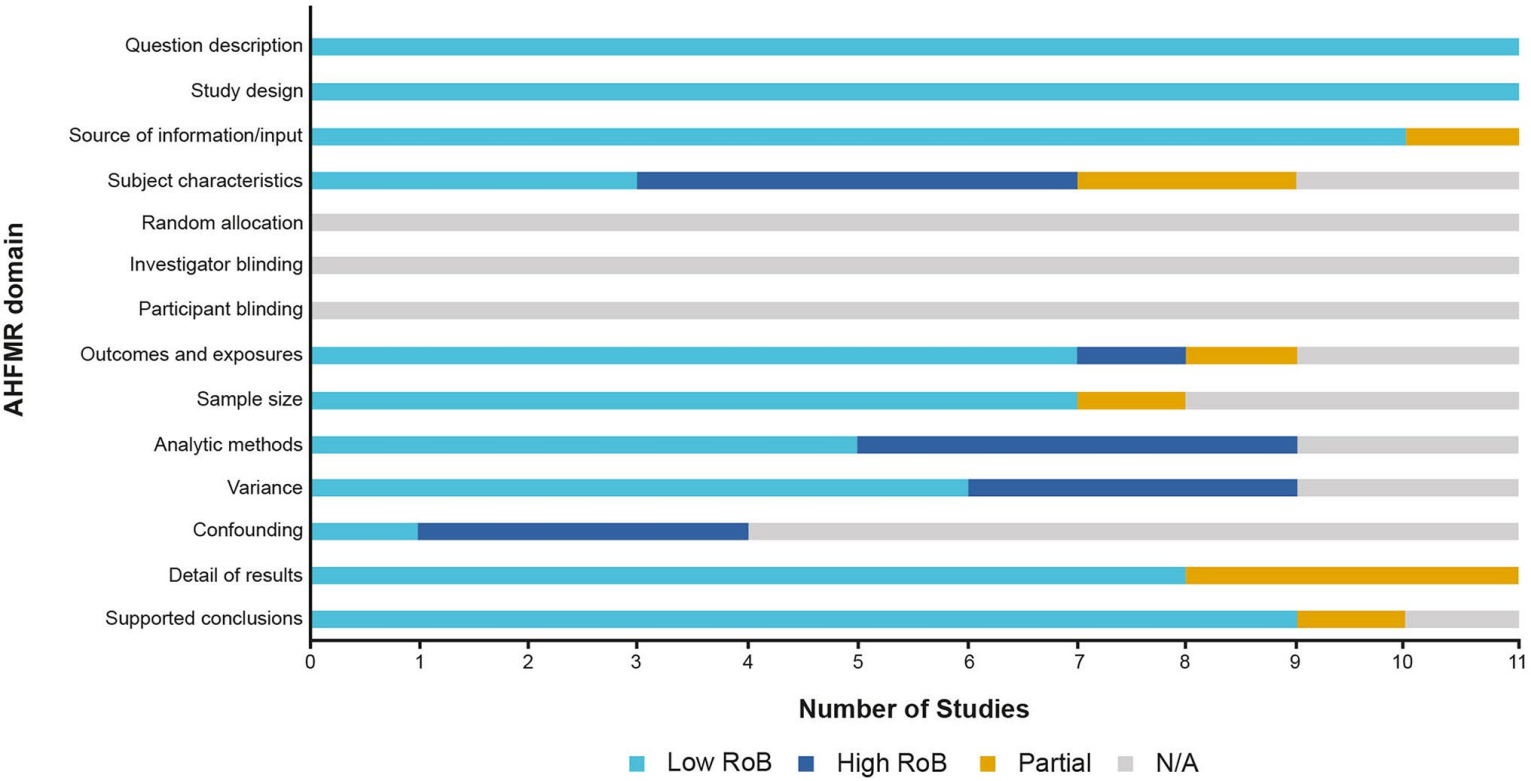
Summary of quality assessments of CRU studies included in the SLR using the AHFMR checklist for quantitative studies. *AHFMR* Alberta Heritage Foundation for Medical Research, *CRU* cost and resource use, *N/A* not applicable, *RoB* risk of bias, *SLR* systematic literature review

Overall, the risk of bias of the 11 included CRU studies was deemed to be low-to-moderate based on the AHFMR checklist. Most studies had a clear objective, detailed and appropriate study design and methodology, clearly described data sources, robust outcome measures and appropriate reporting of results and conclusions. A summary of the quality assessments is presented in Fig. 6 with full details in the Supplementary Appendix.

### Economic evaluations

#### Study characteristics

Characteristics of identified economic evaluations are presented in Table 2. Two studies were conducted from the perspective of the US [28, 30], one from China [35] and one from South Africa [39]. Three studies evaluated healthcare system perspectives [28, 35, 39], while one evaluated health payer and societal perspectives [30].

**Table 2:** Characteristics of included economic evaluations

Study name	Country	Perspective	Population	Compared intervention	Source data	Type of evaluation	Time horizon	Discount rate
ICER 2020	US	Healthcare system perspective in which third-party insurers reimburse hospitalisations through bundled payments	Hospitalised patients with COVID-19 split into the following subgroups: moderate-to-severe cases; mild cases	Remdesivir + SoC vs. SoC alone	Clinical data: ACTT-1, NCT04292730, RECOVERY, SOLIDARITY, US epidemiological evidence, US life tables, Pickard 2019. Utility data: Smith & Roberts 2002, Barbut 2019, Sullivan & Ghushchyan 2006. Costs were sourced from Rae 2020, the Redbook, Gilead press release	Cost-utility	Lifetime time horizon with a 1-month cycle length	3% per year
Jiang 2021	China	Healthcare system perspective	Hospitalised patients with COVID-19	5-day course of remdesivir to severe-state patients and SoC to mild-to-moderate patients vs. SoC to patients of any severity	Cost inputs: Wise 2020, Ma 2020, and Li 2020. The number of severe patients that become critical: WHO COVID-19 transmission questions. RT-PCR test fees: based on the official charge in Hubei province. Health utility values: 2010 WHO global burden of disease, Yang 2017, Zhou 2012. Efficacy data: Jiang 2021. Epidemic parameters: He 2020, Linton 2020, The Novel Coronavirus Pneumonia Emergency Response Epidemiology Team 2020, Collman 2020, Yang 2020, Miyamae 2020, Jiang 2020, Li 2020, and Pan 2020	Cost-utility	January to March 2020 (55-day period)	5% per year
Jo 2021	South Africa	Healthcare system perspective	Ventilated and non-ventilated COVID-19 patients in the ICU	Dexamethasone to ventilated patients and remdesivir to non-ventilated patients vs. SoC	Cost inputs: National Institute for Communicable Disease COVID-19 guidelines, Anadolu Agency, Davies 2020 SLR, Business Insider South Africa, OpenData South Africa. Clinical-Mortality data: WHO-Solidarity, RECOVERY and ACTT-1 trials	Cost-effectiveness	August 2020 to January 2021	Capital assets discounted at a 5% annual rate
Sheinson 2021	US	Health payer perspective, which accounted for short-term bundled payments in the hospital and long-term healthcare system costs Societal perspective, including productivity losses due to premature COVID-19-related mortality FFS, including drug costs and per diem hospital payments instead of bundled costs	Patients hospitalised with COVID-19	Treatment arm (consisting of remdesivir + dexamethasone) vs. BSC	Clinical inputs: ACTT-1, RECOVERY and WHO-SOLIDARITY trials, US CDC, and Lone 2016. Health utilities: Sullivan 2006, Padula 2020, Herridge 2011 and Ara 2008. Societal costs: Grosse 2019. Healthcare system costs: calculated based on the published literature	Cost-utility	Lifetime model including a short-term decision tree followed by a Markov model with an annual cycle length and half-cycle correction	3% per year

*ACTT-1* adaptive COVID-19 treatment trial, *BSC* best supportive care, *CDC* US Centers for Disease Control and Prevention, *FFS* fee-for-service, *ICER* Institute for Clinical and Economic Review, *ICU* intensive care unit, *RT-PCR* reverse transcription polymerase chain reaction, *SLR* systematic literature review, *SoC* standard of care, *WHO* World Health Organization

Two studies evaluated all hospitalised patients with COVID-19 [30, 35], while the other two evaluated hospitalised patients with COVID-19 according to subgroups [28, 39]. ICER 2020 separately evaluated moderate-to-severe and mild cases, with severe disease assumed to be consistent with the criteria in ACTT-1, and Jo 2021 evaluated subgroups of ventilated and non-ventilated patients in the ICU [39].

Three studies used a cost-utility model and reported incremental cost-effectiveness ratios [28, 30, 35], while one study used a cost-effectiveness model, reporting incremental cost-effectiveness ratios in terms of costs per death averted [39]. ICER 2020 compared remdesivir + standard of care (SoC) to SoC alone. Dexamethasone was included in SoC in the moderate-to-severe population [28]. Jiang 2021 assigned remdesivir + SoC to all severe-state patients and SoC alone to moderate-to-mild patients [35]. Sheinson 2021 compared potential treatments including remdesivir and dexamethasone with best supportive care [30]. Jo 2021 compared treatment with remdesivir for non-ventilated and dexamethasone for ventilated patients with SoC [39].

Various assumptions about the benefit of remdesivir were employed in the economic analyses using inputs from various key data sources driving the respective results. ICER 2020 assumed remdesivir has no survival benefit, but has benefits in reducing the progression of patients to requiring high-flow oxygen, non-invasive ventilation, mechanical ventilation, and ECMO, using final ACTT-1 results based on the overall population [28]. Jiang 2021 used results from a meta-analysis to assume an odds ratio of 1.81 for clinical improvement with remdesivir [35]. Jo 2021 used ACTT-1 trial data to assume the benefit of remdesivir as decreasing the average ICU LOS from 15 to 10 days [39]. Finally, Sheinson 2021 used an average of final ACTT-1 trial results for remdesivir and RECOVERY trial results for dexamethasone and estimated a 23% reduction in the hazard of progression to mechanical ventilation, a 10% to 25% increase in speed to recovery, and a 0% to 33% reduction in hazards of mortality for the combined remdesivir + dexamethasone treatment [30].

#### Study results

**Fig. 7:**
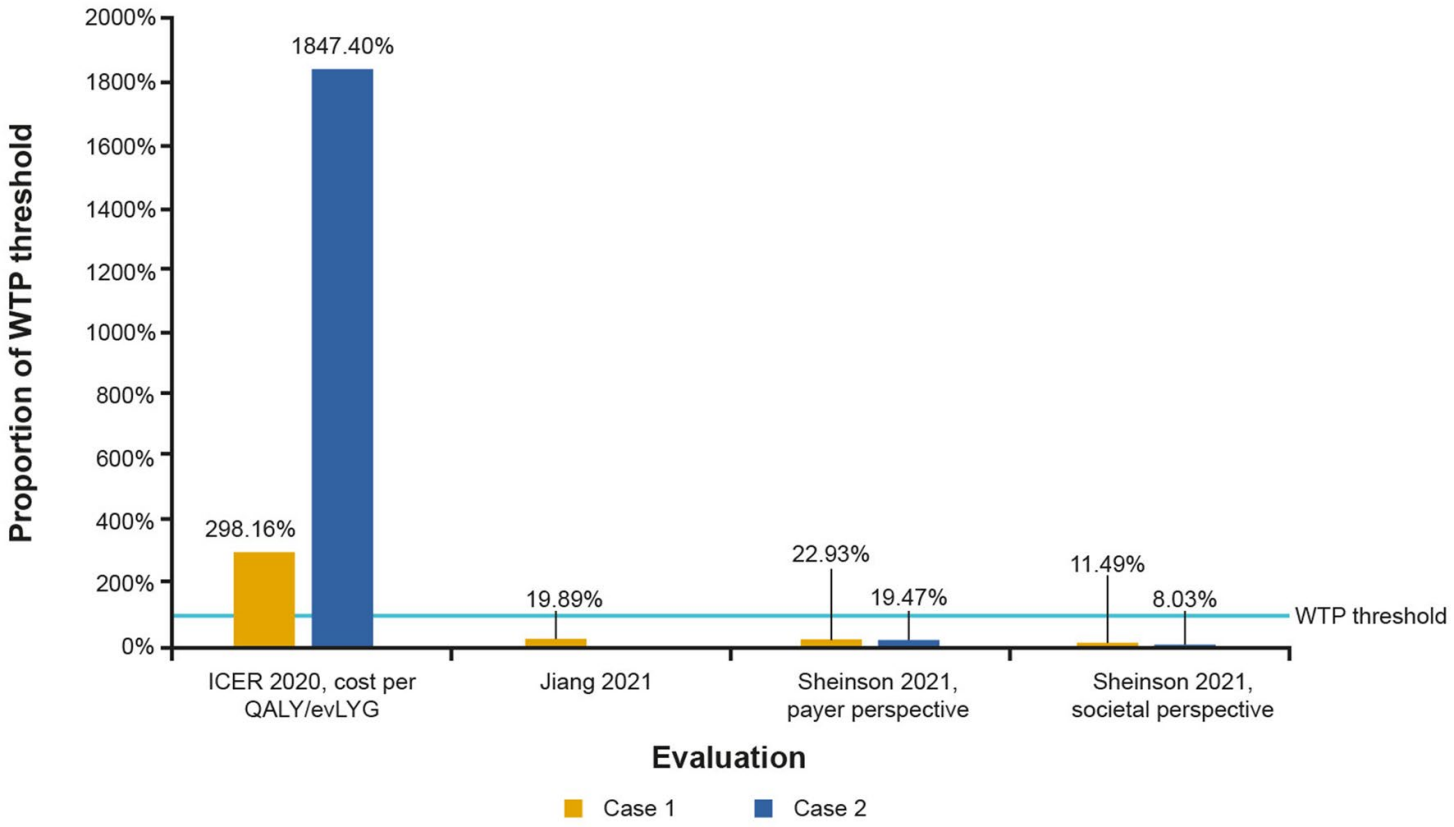
Cost per QALY across studies in terms of the WTP threshold of the respective country. WTP thresholds were taken as $100,000 for the US (ICER 2020, Sheinson 2021) and CN¥ 70,892 for China (Jiang 2021). ICER 2020: Case 1, moderate-to-severe patients; Case 2, mild patients. Jiang 2021: Case 1, remdesivir administered to severe patients only. Sheinson 2021, payer perspective: Case 1, bundled payment; Case 2, FFS. Sheinson 2021, societal perspective: Case 1, bundled payment; Case 2, FFS. *CN¥* Chinese Yuan, *FFS* fee-for-service, *ICER* Institute for Clinical and Economic Review, *QALY* quality-adjusted life-years, *WTP* willingness-to-pay

Three economic evaluations reported costs per QALY associated with remdesivir treatment models. The results are presented in **Fig. ****7** as percentages of the willingness-to-pay (WTP) thresholds of the relevant country. Two models reported costs per QALYs that were well within the WTP threshold. A US-based cost-utility model reported by Sheinson 2021 investigated results for a treatment regimen that included remdesivir and dexamethasone. Accounting for the payer perspective only, costs per QALY were $22,933 and $19,469 for bundled and FFS payments, respectively (22.93% and 19.47% of WTP of $100,000, respectively). When additionally accounting for the societal perspective through the inclusion of productivity losses, the study found further improved costs per QALYs of $11,492 and $8,028 for bundled and FFS payments, respectively (11.49% and 8.03% of WTP of $100,000, respectively) [30]. Jiang 2021 assessed a cost-utility model from the perspective of China, in which remdesivir was only administered to patients progressing to severe COVID-19 and estimated a cost per QALY of CN¥ 14,098 (approximately $2,218; 19.89% of WTP of CN¥ 70,892) [35].

However, the US-based ICER 2020 found the cost per QALY of remdesivir in addition to SoC to be $298,160 (298.16% of WTP of $100,000) in moderate-to-severe cases and $1,847,400 in mild cases, substantially higher than the WTP threshold and considerably higher than the results reported in other economic evaluations [28]. In this model, a key cost-driver was the acquisition cost of remdesivir despite cost offsets associated with fewer people on remdesivir progressing to higher levels of respiratory support. In ICER 2020, cost-effectiveness price benchmarks for the average treatment course of remdesivir were calculated for different WTP thresholds. For a threshold of $50,000 per QALY, the price benchmark was estimated as $2,470 for moderate-to-severe cases and $70 for mild cases. For a threshold of 100,000 per QALY, the price benchmark was $2,770 and $150, respectively, for moderate-to-severe and mild cases [28].

Finally, in a cost-effectiveness study, Jo 2021 estimated the impact of administering remdesivir to non-ventilated patients only and found that this intervention would be strongly dominant compared to SoC, with 26 deaths averted. An additional scenario in which remdesivir was administered to non-ventilated and dexamethasone was administered to ventilated patients was also strongly dominant, with 408 deaths averted and $14,591 saved. Conversely, the scenarios where dexamethasone was administered without remdesivir resulted in cost increases but were cost-effective when contrasted against the South African threshold value for deaths averted ($36,000) [39].

#### Study quality

**Fig. 8:**
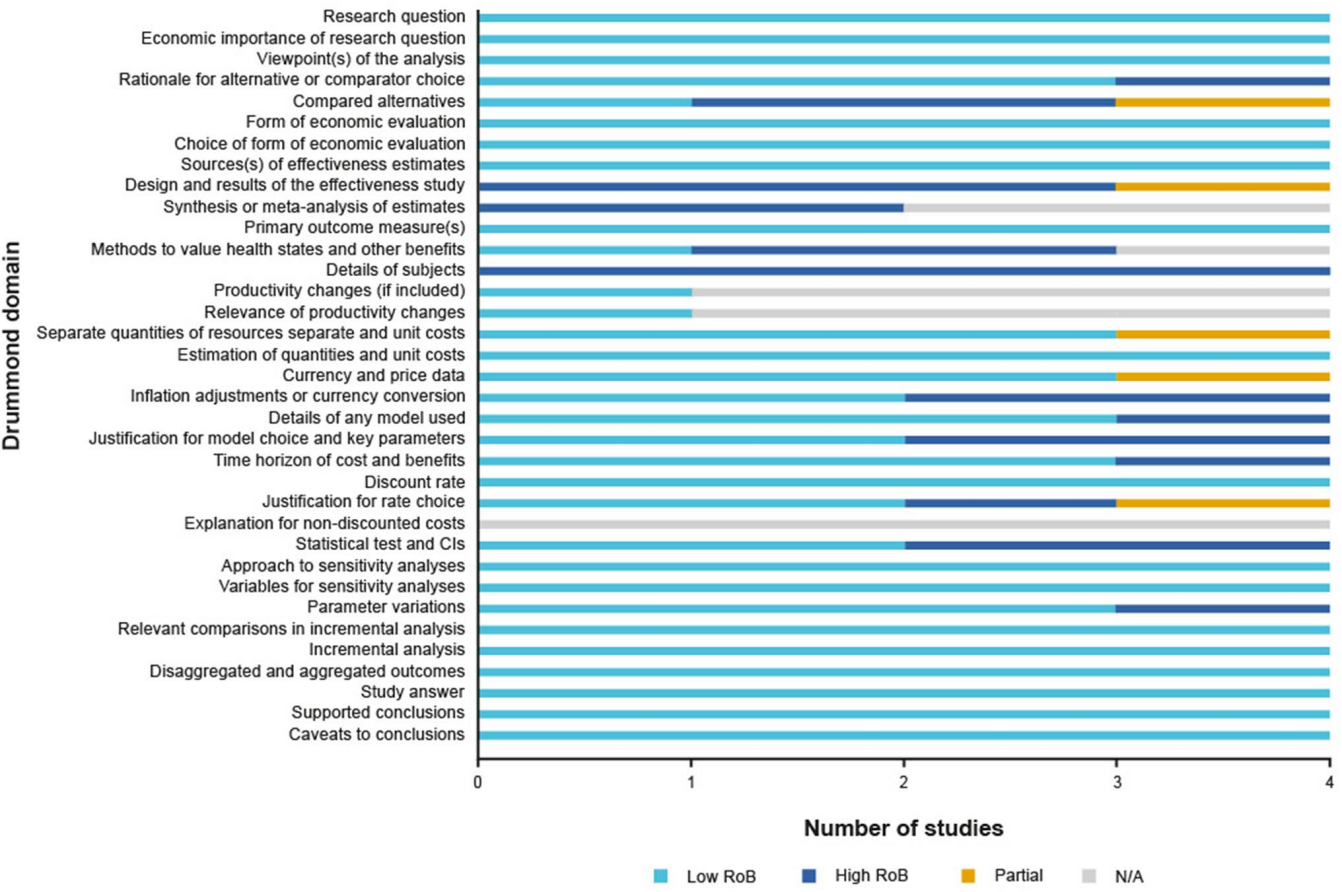
Summary of quality assessments of economic evaluations included in the SLR using the Drummond checklist. *CI* confidence interval, *N/A* not applicable, *RoB* risk of bias, *SLR* systematic literature review

The quality of the included economic evaluations was deemed to be moderate-to-high based on the Drummond checklist. The studies performed well in the majority of study design and analysis and interpretation of results domain questions. Performance in the data collection domain was more varied with some studies lacking in the reporting of details of the effectiveness study used and methods of synthesis/meta-analysis and valuation of health states methods. A summary of quality assessments is presented in Fig. 8 with full details in the Supplementary Appendix.

## Discussion

Overall, the current economic evidence relating to remdesivir in the COVID-19 pandemic is somewhat limited, with only eight studies reporting resource use, five reporting cost and four economic evaluations of moderate-to-high quality. Nonetheless, the identified studies reported data that are particularly useful when considering the wider clinical context for use of remdesivir. Shorter hospital LOS and a reduction in the need for additional rehabilitation post-hospitalisation reported by Anderson 2021 highlight the potential of remdesivir for relieving pressures on the healthcare system, with findings consistent across different age groups [27]. Similarly, the findings from Béraud 2020 and Soriano 2021 further support reduced healthcare burden with remdesivir use through reductions in bed occupancy in France and Spain, respectively [32, 34]. However, Anderson 2021 noted that while remdesivir treatment resulted in shorter LOS for some, a proportion of patients may have a prolonged hospital stay in order to complete the clinically accepted 5-day course of remdesivir [27]. This highlights the need for further studies to explore the balance between the length of remdesivir treatment course and savings in hospital resource use.

While not captured in this SLR, key benefits of remdesivir to LOS and mortality have been reported in several high-profile trials and large observational studies [19, 40–43]. ACTT-1 reported a 5-day decrease in duration of hospitalisation in the remdesivir compared to the placebo group [19]. However, in contrast, the randomised European DisCoVeRy trial (a sub-study of the SOLIDARITY trial) compared remdesivir with SoC in a cohort of 857 patients and found no significant difference in LOS (p = 0.49) [44]. The discrepancy in the results highlights the critical importance of considering the characteristics of the patient population and the timing of remdesivir prescription and is highlighted when focussing more closely on the results for specific subgroups of patients in these trials. For example, in DisCoVeRy, in the subgroup of patients that did not require mechanical ventilation (MV) or ECMO at baseline, remdesivir significantly reduced progression to MV or ECMO [44]. Furthermore, in ACTT-1, additional analyses showed that patients who received remdesivir within less than 6 days from symptom onset had shorter LOS by 14 days compared with patients receiving placebo [19]. This reflects that, as an antiviral, remdesivir is most effective when prescribed early in infection when viral replication is high. It also has implications for the economic evaluations that used ACTT-1 data to inform their analyses, in that the true efficacy of remdesivir for its intended use may be underestimated. The benefit of early remdesivir initiation is also supported by interventional and observational studies that have reported it to be associated with improved recovery, lower rates of ICU admission and reduced mortality [45–47].

In line with this, studies identified in this SLR reported earlier administration of remdesivir over the course of the pandemic (Garcia-Vidal 2021 and Mozaffari 2021 [31, 43]) and an increase in prescriptions, particularly in combination with dexamethasone (Bechman 2021 and Garcia-Vidal 2021 [31, 33]), reflective of clinicians’ increasing experience with remdesivir.

Three out of four economic evaluations found that the use of remdesivir under various scenarios would be either cost-effective or strongly dominant [30, 35, 39]. The report from ICER 2020 was the only exception, finding a cost-ineffective result at up to 1,800% of the WTP threshold in the US [28]. However, the ICER 2020 report has received considerable criticism from the scientific community based on several important limitations in the methodology employed in the model and as a result, underestimation of the value of remdesivir [30, 48]. First, by nature of the ICER model adopting a healthcare system perspective, it does not consider broader societal benefits and costs in its cost-effectiveness model and, therefore, does not take into account the impact of income loss resulting from the pandemic. Furthermore, in its cost recovery model, the assumption of zero costs for research and development is not an accurate reflection of the real-life scenario. As such, the cost recovery price assessments may underestimate the true price of remdesivir as they do not account for any additional factors such as innovation, value, and revenue generation.

The results of the economic evaluations must be interpreted in the context of the clinical scenarios and country-specific considerations. For example, each study assessed a different patient population with different disease characteristics, ranging from mild-to-severe illness. Similarly, the results of each study are dependent on the healthcare systems in place in each country that studies originated in, ranging from National Health Service structures (UK and Spain) to statutory insurance (France) and a pure market (US) in this SLR. Furthermore, resources were costed differently across studies, with some using national tariffs, for example, and others using insurance claims. This may impact the interpretation of analyses. Nevertheless, three evaluations reported cost-effective or strongly dominant results, indicating that remdesivir may be a beneficial treatment regardless of specific patient characteristics or country-specific healthcare systems.

The strengths of this review include the robust, systematic approach, with comprehensive search strategy that included multiple literature sources. Publications from all countries were considered and the quality of the included articles was appraised using published, validated tools [24, 25]. Furthermore, although quality is less assured, the consideration of non-peer-reviewed articles from MedRxiv and the Social Science Research Network allowed for the full breadth of emerging evidence on COVID-19 to be considered.

However, there are also several limitations. First, despite the high volume of evidence that continues to be published, few have investigated economic outcomes for remdesivir, meaning that the majority of conclusions are based on one or a small number of studies. For example, the consistency in findings from the French and Spanish CRU studies included in this SLR may be explained in part by the fact that both studies used the results of ACTT-1 to estimate the impact of remdesivir on LOS and oxygen requirement. In addition, the identified studies include heterogenous populations, making it difficult to draw comparisons or comment on the most reliable results. Many included studies also relied on modelling estimates to draw CRU conclusions; as such, further studies with real-world evidence are needed to confirm and strengthen the results identified in this SLR. Specifically, within the evidence base, Béraud 2020 used diagnosis-related group (DRG)-based national hospital tariffs to estimate the daily cost of inpatient care in conventional and ICU wards. However, it is unclear whether the cost of remdesivir was included within the DRG tariffs (as is standard practice for DRG-based systems) or calculated in addition to the DRG tariff, which may have impacted cost-effectiveness results [34]. Further, Sheinson 2021 used a lifetime model for COVID-19, a largely acute disease [30]. While COVID-19 symptoms can persist in some patients for weeks or months after initial infection, referred to as “long COVID-19” [49], it is unclear whether a model extending through a patient’s lifetime provides a realistic representation of the disease.

Another important consideration is that, due to the nature of the early stages of the pandemic, most evidence included in this SLR focussed on older adults with the initial strain of the virus [50]. However, a shift in the demography of the pandemic continues to emerge, with increasing numbers of cases reported in younger adults and higher prevalence of different variants, including Delta and Omicron, as well as a shift in the disease pattern from a pandemic to a seasonal endemic [51–53]. Nonetheless, findings of this SLR, covering a variety of geographies and patient populations still provide a useful basis and overview of the initial evidence base, and can be used to inform future HTA processes as well as setting the foundations for future studies.

Several additional studies have been published since this review was conducted, reporting remdesivir-related economic evaluations and cost data from a variety of countries. A further cost-consequence study from the perspective of the US [54] and a cost-effectiveness study from Turkey [55] have reported that remdesivir would be a cost-effective and strongly dominant treatment for COVID-19, respectively. In addition, epidemiological modelling studies from Germany [56], South Africa [57], Saudi Arabia [58] and Portugal [59] have reported that remdesivir administration would result in significant increases in hospital bed capacities, while a cohort study in Hong Kong has found that early remdesivir treatment is associated with significantly shorter hospital LOS [60]. Of note, several of these studies reported reduced ICU LOS or increased ICU bed capacity with remdesivir, in line with Béraud 2020 and Soriano 2021, a notable outcome considering the substantially higher costs associated with ICU stays in comparison with general ward stays [32, 34].

## Conclusions

Findings of this SLR suggest that patient population and timing are two essential factors to consider in clinical practice for treatment decisions regarding remdesivir. Further investigations are necessary to characterise the treatment needs of a new patient population with the shifting state of the pandemic. However, despite changes in patient populations, COVID-19 treatments that can minimise hospital LOS will continue to be highly important, as breakthrough infections in vaccinated individuals, and limitations to widespread vaccinations will continue to drive COVID-19 infections. As such, hospitals may continue experiencing surges in patients with seasonal waves of the virus, necessitating treatments that alleviate pressures on hospital capacities and healthcare systems.

## Supplementary Information

Below is the link to the electronic supplementary material.

Supplementary file1 (DOCX 146 KB)

## Abbreviations

ACTT-1: Adaptive COVID-19 treatment trial
AHFMR: Alberta Heritage Foundation for Medical Research
CN¥: Chinese Yuan
CRU: Cost and resource use
ECMO: Extracorporeal membrane oxygenation
FDA: Food and Drug Administration
FFS: Fee-for-service
HTA: Health Technology Assessment
ICER: Institute for Clinical and Economic Review
ICU: Intensive care unit
IMV: Invasive mechanical ventilation
LOS: Length of stay
MV: Mechanical ventilation
PRISMA: Preferred Reporting Items for Systematic Reviews and Meta-Analyses
QALY: Quality-adjusted life-years
SARS-CoV-2: Severe acute respiratory syndrome coronavirus 2
SLR: Systematic literature review
SoC: Standard of care
WTP: Willingness-to-pay
