# Supplementary material for: Remdesivir-related cost-effectiveness and cost and resource use evidence in COVID-19: a systematic review
Source: Infection. 2022 Oct 12;51(2):285–303. doi: 10.1007/s15010-022-01930-8 (PMC9555695; doi:10.1007/s15010-022-01930-8)
Supplement: Supplementary file 1 — Supplementary file1 (DOCX 146 KB) [file 15010_2022_1930_MOESM1_ESM.docx]

**Remdesivir-Related Cost-Effectiveness and Cost and Resource Use Evidence in COVID-19: A Systematic Review**

Molly Murton,^1^ Emma Drane,^2^ James Jarrett,^3^ Oliver A. Cornely,^4^ Alex Soriano^5^

^1^Costello Medical, London, UK;

^2^Costello Medical, Cambridge, UK;

^3^Gilead Sciences, Inc. London, UK;

^4^University of Cologne, Faculty of Medicine and University Hospital Cologne, Department I of Internal Medicine, Excellence Centre for Medical Mycology (ECMM), Cologne, Germany; University of Cologne, Faculty of Medicine and University Hospital Cologne, Cologne Excellence Cluster on Cellular Stress Responses in Aging-Associated Diseases (CECAD), Cologne, Germany; University of Cologne, Faculty of Medicine and University Hospital Cologne, Clinical Trials Centre Cologne (ZKS Köln), Cologne, Germany; German Centre for Infection Research (DZIF), Partner Site Bonn-Cologne, Cologne, Germany;

^5^Department of Infectious Diseases, Hospital Clínic of Barcelona, University of Barcelona, IDIBAPS.

**SUPPLEMENTARY DATA**

**Table 3. Search terms for MEDLINE (searched via Ovid SP)**

| **Dates searched: Original, 29th July 2020; Update 1, 24th May 2021**  **Records retrieved: Original, 2,139; Update 1, 95** | | | | |
| --- | --- | --- | --- | --- |
| **Term group** | **#** | **Search terms** | **Results 29.07.20** | **Results 24.05.21** |
| COVID-19 | 1 | exp coronavirus/ or exp coronavirus infections/ | 28963 | 96521 |
|  | 2 | SARS-CoV-2.ti,ab,kf. | 10805 | 44062 |
|  | 3 | (coronavirus$ or corona virus$).ti,ab,kf. | 26613 | 66220 |
|  | 4 | (Covid-19 or Covid19).ti,ab,kf. | 32951 | 120968 |
|  | 5 | nCoV.ti,ab,kf. | 1051 | 1895 |
|  | 6 | or/1-5 | 56003 | 156358 |
| Study design: Economic evaluations | 7 | Cost-benefit analysis/ | 81222 | 84636 |
|  | 8 | "Costs and cost analysis"/ | 48710 | 49551 |
|  | 9 | Economics/ | 27208 | 27326 |
|  | 10 | (cost$ adj (effective$ or utilit$ or consequence$ or benefit$ or minimi$)).ti,ab,kf. | 148921 | 159617 |
|  | 11 | (economic evaluation$ or economic analysis or life year$ gained or ICER or QALY$ or DALY$ or quality adjusted or adjusted life year$ or disability adjusted life or qald$ or qale$ or qtime$).ti,ab,kf. | 34861 | 37826 |
|  | 12 | Quality-adjusted life years/ | 12295 | 13272 |
|  | 13 | Value of life/ | 5706 | 5746 |
|  | 14 | or/7-13 | 271566 | 285078 |
| Study design utilities and HRQoL | 15 | (health utilit$ or health state$1 or illness state$1 or HSUV or HSUVs or health state$ value$ or health state$ preference$ or utility assessment$ or utility measure$ or preference based or utility based).ti,ab,kf. | 9718 | 10497 |
|  | 16 | ((index adj3 wellbeing) or (quality adj3 wellbeing) or qwb).ti,ab,kf. | 756 | 841 |
|  | 17 | (multiattribute$ or multi attribute$).ti,ab. | 881 | 968 |
|  | 18 | utility.ab. /freq=2 | 17665 | 19199 |
|  | 19 | (utilities or disutilit$).ti,ab,kf. | 7515 | 8097 |
|  | 20 | (euro qual or euro qual5d or euro qol5d or eq-5d or eq5-d or eq5d or eq 5d or euroqual or euroqol or euro qol or euroqual5d or euroqol5d or eq-sdq or eqsdq).ti,ab,kf. | 11260 | 12709 |
|  | 21 | (short form$ or shortform$).ti,ab. | 33662 | 36413 |
|  | 22 | (sf36$ or sf 36$ or sf thirtysix or sf thirty six).ti,ab,kf. | 22263 | 23463 |
|  | 23 | (sf6 or sf 6 or sf6d or sf 6d or sf six D or sfsixD or sf six or sfsix or sf8 or sf 8 or sf eight or sfeight).ti,ab,kf. | 3319 | 3485 |
|  | 24 | (sf12 or sf 12 or sf twelve or sftwelve).ti,ab,kf. | 4809 | 5219 |
|  | 25 | (sf16 or sf 16 or sf sixteen or sfsixteen).ti,ab,kf. | 30 | 30 |
|  | 26 | (sf20 or sf 20 or sf twenty or sftwenty).ti,ab,kf. | 336 | 343 |
|  | 27 | (15D or 15-D or 15 dimension).ti,ab,kf. | 5279 | 5558 |
|  | 28 | visual analog$ scale$.ti,ab,kf. | 55753 | 60542 |
|  | 29 | (standard gamble$ or sg).ti,ab,kf. | 10860 | 11763 |
|  | 30 | (time trade off$1 or time tradeoff$1 or tto or timetradeoff$1).ti,ab,kf. | 1916 | 2026 |
|  | 31 | (health$1 year$1 equivalent$1 or hye or hyes).ti,ab,kf. | 78 | 82 |
|  | 32 | (hui or hui1 or hui2 or hui3 or rosser).ti,ab,kf. | 1635 | 1748 |
|  | 33 | *quality of life/ and (quality of life or qol or hrqol).ti,ab,kf. | 73107 | 79263 |
|  | 34 | quality of life/ and ((quality of life or qol) adj3 (improv$ or chang$)).ti,ab,kf. | 27577 | 30415 |
|  | 35 | quality of life/ and ((quality of life or qol or hrqol) adj (score$1 or measure$1)).ti,ab,kf. | 13898 | 14906 |
|  | 36 | quality of life/ and health-related quality of life.ti,ab,kf. | 32132 | 35181 |
|  | 37 | quality of life/ and ec.fs. | 10087 | 10543 |
|  | 38 | quality of life/ and (health adj3 status).ti,ab,kf. | 9027 | 9676 |
|  | 39 | ((qol or hrqol or quality of life).ti,kf. or *quality of life/) and ((qol or hrqol$ or quality of life) adj2 (increas$ or decrease$ or improv$ or declin$ or reduc$ or high$ or low$ or effect or effects or worse or score or scores or change$1 or impact$1 or impacted or deteriorat$)).ab. | 38039 | 41979 |
|  | 40 | or/15-39 | 239637 | 260370 |
| Study design: Cost and resource use studies | 41 | Cost allocation/ | 2004 | 2008 |
|  | 42 | Cost control/ | 21498 | 21580 |
|  | 43 | Cost savings/ | 11845 | 12213 |
|  | 44 | Cost of illness/ | 27263 | 28806 |
|  | 45 | Cost sharing/ | 2525 | 2599 |
|  | 46 | "Deductibles and coinsurance"/ | 1750 | 1782 |
|  | 47 | Medical savings accounts/ | 535 | 542 |
|  | 48 | Health care costs/ | 39622 | 41309 |
|  | 49 | Direct service costs/ | 1190 | 1205 |
|  | 50 | Drug costs/ | 16092 | 16584 |
|  | 51 | Employer health costs/ | 1092 | 1094 |
|  | 52 | Hospital costs/ | 11067 | 11474 |
|  | 53 | Health expenditures/ | 20289 | 21390 |
|  | 54 | Capital expenditures/ | 1989 | 1996 |
|  | 55 | exp economics, Hospital/ | 24561 | 25109 |
|  | 56 | exp economics, Medical/ | 14206 | 14261 |
|  | 57 | Economics, nursing/ | 3999 | 4003 |
|  | 58 | Economics, pharmaceutical/ | 2941 | 2990 |
|  | 59 | exp "Fees and charges"/ | 30328 | 30703 |
|  | 60 | exp Budgets/ | 13716 | 13826 |
|  | 61 | Financial management/ | 16548 | 16679 |
|  | 62 | (low adj cost).mp. | 58974 | 65725 |
|  | 63 | (high adj cost).mp. | 14761 | 16016 |
|  | 64 | (health?care adj cost$).mp. | 11789 | 13089 |
|  | 64 | (fiscal or funding or financial or finance).ti,ab,kf. | 149872 | 162616 |
|  | 66 | (cost adj estimate$).mp. | 2287 | 2423 |
|  | 67 | (cost adj variable$).mp. | 163 | 172 |
|  | 68 | (unit adj cost$).mp. | 2536 | 2699 |
|  | 69 | (economic$ or pharmacoeconomic$ or price$ or pricing).ti,ab,kf. | 324109 | 352144 |
|  | 70 | ((resource$ or healthcare$ or service$) adj3 (use$ or utilis$ or utiliz$ or consume$ or consuming or consumption$)).ti,ab,kf. | 104535 | 113997 |
|  | 71 | ((patient$ or caregiver$ or carer$ or social$ or society$ or family$) adj2 (burden$ or productiv$)).ti,ab,kf. | 15873 | 17819 |
|  | 72 | ("length of stay" or utili?ation or "economic burden" or "cost-of-illness" or nursing cost$ or physician cost$ or physician visit$ or "out of pocket").ti,ab,kf. | 276201 | 299320 |
|  | 73 | (absenteeism or presenteeism or employment or unemployment).ti,ab,kf. or exp presenteeism/ or exp absenteeism/ or exp unemployment/ or exp employment/ | 142377 | 149960 |
|  | 74 | or/41-73 | 1074391 | 1154290 |
| Exclusion terms | 75 | exp Animals/ not exp Humans/ | 4720834 | 4832148 |
|  | 76 | (editorial or "case reports").pt. | 2647466 | 2745845 |
|  | 77 | (case stud$ or case report$).ti. | 297771 | 318779 |
|  | 78 | or/75-77 | 7400884 | 7613270 |
| Combination for original SLR | 79 | 6 and (14 or 40 or 74) | 3154 | N/A |
|  | 80 | 79 not 78 | 2550 | N/A |
|  | 81 | limit 80 to yr=”2019-current” | 2139 | N/A |
| Remdesivir* | 82 | (remdesivir or RDV or veklury or GS-5734 or GS5734).af. | N/A | 1792 |
| Combination for SLR update | 83 | 6 and (14 or 40 or 74) and 79 | N/A | 105 |
|  | 84 | 80 not 78 | N/A | 95 |
|  | 85 | limit 81 to yr="2019-current" | N/A | 95 |

**Databases:** Ovid MEDLINE(R) and Epub Ahead of Print, In-Process & Other Non-Indexed Citations and Daily 1946 to 27th July 2020 and 21st May 2021.

**Footnotes:** *Remdesivir terms were not used in the original SLR because the scope of the review was originally broader, but were added to narrow the scope to only include articles considering remdesivir for the SLR update.

**Abbreviations:** HRQoL: health-related quality-of-life; N/A: not applicable; SLR: systematic literature review.

**Table 4. Search terms for Embase (searched via Ovid SP)**

| **Dates searched: Original, 29th July 2020; Update 1, 24th May 2021**  **Records retrieved: Original, 2,516; Update 1, 266** | | | | |
| --- | --- | --- | --- | --- |
| **Term group** | **#** | **Search terms** | **Results 29.07.20** | **Results 24.05.21** |
| COVID-19 | 1 | exp coronavirinae/ or exp coronavirus infection/ | 25589 | 143461 |
|  | 2 | SARS-CoV-2.ti,ab,kw. | 10198 | 41568 |
|  | 3 | (coronavirus$ or corona virus$).ti,ab,kw. | 27032 | 64302 |
|  | 4 | (Covid-19 or Covid19).ti,ab,kw. | 31833 | 116822 |
|  | 5 | nCoV.ti,ab,kw. | 1007 | 1875 |
|  | 6 | or/1-5 | 58774 | 163475 |
| Study design: Economic evaluations | 7 | Cost benefit analysis/ or exp economic evaluation/ or cost effectiveness analysis/ or cost minimization analysis/ | 306617 | 318990 |
|  | 8 | Economics/ or health economics/ or socioeconomics/ or economic aspect/ or pharmacoeconomics/ | 496561 | 509683 |
|  | 9 | (cost$ adj (effective$ or utilit$ or consequence$ or benefit$ or minimi$)).ti,ab,kw. | 207495 | 221828 |
|  | 10 | (economic evaluation$ or economic analysis or life year$ gained or ICER or QALY$ or DALY$ or quality adjusted or adjusted life year$ or disability adjusted life or qald$ or qale$ or qtime$).ti,ab,kw. | 53948 | 58328 |
|  | 11 | quality adjusted life year/ | 26712 | 28934 |
|  | 12 | or/7-11 | 837672 | 870403 |
| Study design: Utilities and HRQoL | 13 | (health utilit$ or health state$1 or illness state$1 or HSUV or HSUVs or health state$ value$ or health state$ preference$ or utility assessment$ or utility measure$ or preference based or utility based).ti,ab,kw. | 16329 | 17629 |
|  | 14 | ((index adj3 wellbeing) or (quality adj3 wellbeing) or qwb).ti,ab,kw. | 1201 | 1337 |
|  | 15 | (multiattribute$ or multi attribute$).ti,ab. | 1125 | 1219 |
|  | 16 | utility.ab. /freq=2 | 27586 | 29921 |
|  | 17 | (utilities or disutilit$).ti,ab,kw. | 12409 | 13346 |
|  | 18 | (euro qual or euro qual5d or euro qol5d or eq-5d or eq5-d or eq5d or eq 5d or euroqual or euroqol or euro qol or euroqual5d or euroqol5d or eq-sdq or eqsdq).ti,ab,kw. | 20920 | 23471 |
|  | 19 | (short form$ or shortform$).ti,ab. | 45908 | 49808 |
|  | 20 | (sf36$ or sf 36$ or sf thirtysix or sf thirty six).ti,ab,kw. | 38222 | 40438 |
|  | 21 | (sf6 or sf 6 or sf6d or sf 6d or sf six D or sfsixD or sf six or sfsix or sf8 or sf 8 or sf eight or sfeight).ti,ab,kw. | 4563 | 4807 |
|  | 22 | (sf12 or sf 12 or sf twelve or sftwelve).ti,ab,kw. | 8264 | 8898 |
|  | 23 | (sf16 or sf 16 or sf sixteen or sfsixteen).ti,ab,kw. | 54 | 56 |
|  | 24 | (sf20 or sf 20 or sf twenty or sftwenty).ti,ab,kw. | 341 | 351 |
|  | 25 | (15D or 15-D or 15 dimension).ti,ab,kw. | 6692 | 6970 |
|  | 26 | visual analog$ scale$.ti,ab,kw. | 79487 | 86184 |
|  | 27 | (standard gamble$ or sg).ti,ab,kw. | 16226 | 17466 |
|  | 28 | (time trade off$1 or time tradeoff$1 or tto or timetradeoff$1).ti,ab,kw. | 2811 | 2986 |
|  | 29 | (health$1 year$1 equivalent$1 or hye or hyes).ti,ab,kw. | 155 | 161 |
|  | 30 | (hui or hui1 or hui2 or hui3 or rosser).ti,ab,kw. | 2450 | 2644 |
|  | 31 | *quality of life/ and (quality of life or qol or hrqol).ti,ab,kw. | 97459 | 105928 |
|  | 32 | quality of life/ and ((quality of life or qol) adj3 (improv$ or chang$)).ti,ab,kw. | 76416 | 82564 |
|  | 33 | quality of life/ and ((quality of life or qol or hrqol) adj (score$1 or measure$1)).ti,ab,kw. | 29748 | 31969 |
|  | 34 | quality of life/ and health-related quality of life.ti,ab,kw. | 58594 | 63781 |
|  | 35 | quality of life/ and (health adj3 status).ti,ab,kw. | 16273 | 17496 |
|  | 36 | ((qol or hrqol or quality of life).ti,kw. or *quality of life/) and ((qol or hrqol$ or quality of life) adj2 (increas$ or decrease$ or improv$ or declin$ or reduc$ or high$ or low$ or effect or effects or worse or score or scores or change$1 or impact$1 or impacted or deteriorat$)).ab. | 57604 | 62670 |
|  | 37 | or/13-36 | 381883 | 413301 |
| Study design: Cost and resource use studies | 38 | Cost control/ | 68434 | 70511 |
|  | 39 | Cost of illness/ | 19210 | 19858 |
|  | 40 | Health care cost/ | 189603 | 198223 |
|  | 41 | Drug cost/ | 77372 | 79779 |
|  | 42 | Hospital cost/ or cost/ or fee/ | 92431 | 94465 |
|  | 43 | exp Budget/ | 29149 | 30497 |
|  | 44 | Financial management/ | 113030 | 115103 |
|  | 45 | health care cost/ | 189603 | 198223 |
|  | 46 | health care financing/ | 13291 | 13496 |
|  | 47 | (low adj cost).mp. | 66672 | 74411 |
|  | 48 | (high adj cost).mp. | 19334 | 21050 |
|  | 49 | (health?care adj cost$).mp. | 20597 | 22757 |
|  | 50 | (fiscal or funding or financial or finance).ti,ab,kw. | 201122 | 221552 |
|  | 51 | (cost adj estimate$).mp. | 3426 | 3663 |
|  | 52 | (cost adj variable$).mp. | 265 | 281 |
|  | 53 | (unit adj cost$).mp. | 4515 | 4803 |
|  | 54 | (economic$ or pharmacoeconomic$ or price$ or pricing).ti,ab,kw. | 399308 | 432461 |
|  | 55 | ((resource$ or healthcare$ or service$) adj3 (use$ or utilis$ or utiliz$ or consume$ or consuming or consumption$)).ti,ab,kw. | 146549 | 159893 |
|  | 56 | ((patient$ or caregiver$ or carer$ or social$ or society$ or family$) adj2 (burden$ or productiv$)).ti,ab,kw. | 26537 | 29756 |
|  | 57 | ("length of stay" or utili?ation or "economic burden" or "cost-of-illness" or nursing cost$ or physician cost$ or physician visit$ or "out of pocket").ti,ab,kw. | 401267 | 436311 |
|  | 58 | (absenteeism or presenteeism or employment or unemployment).ti,ab,kw. or exp presenteeism/ or exp absenteeism/ or exp unemployment/ or exp employment/ | 146544 | 156556 |
|  | 59 | or/38-58 | 1554291 | 1667603 |
| Exclusion terms | 60 | exp Animal/ not exp Human/ | 4656728 | 4783859 |
|  | 61 | (editorial or "case reports").pt. | 660365 | 691016 |
|  | 62 | (case stud$ or case report$).ti. | 363354 | 389505 |
|  | 63 | or/61-63 | 5667316 | 5850580 |
| Combination | 64 | 6 and (12 or 37 or 59) | 4451 | N/A |
|  | 64 | 64 not 63 | 3726 | N/A |
|  | 66 | limit 65 to yr=2019-current | 2516 | N/A |
| Remdesivir* | 67 | (remdesivir or RDV or veklury or GS-5734 or GS5734).af. | N/A | 4149 |
| Combination for SLR update | 68 | 6 and (12 or 37 or 59) and 64 | N/A | 288 |
|  | 69 | 65 not 63 | N/A | 266 |
|  | 70 | limit 66 to yr="2019-current" | N/A | 266 |

**Database:** Embase 1974 to 27th July 2020 and 21st May 2021.

*Remdesivir terms were not used in the original SLR because the scope of the review was originally broader, but were added to narrow the scope to only include articles considering remdesivir for the SLR update.

**Abbreviations:** HRQoL: health-related quality-of-life; N/A: not applicable; SLR: systematic literature review.

**Table 5. Search terms for the international HTA database (via INAHTA)**

| **Dates searched: Original, NA; Update 1, 24th May 2021**  **Records retrieved: Original, NA; Update 1, 2** | | | | |
| --- | --- | --- | --- | --- |
| **Term group** | **#** | **Search terms** | **Results N/A** | **Results 24.05.21** |
| COVID-19 | 1 | (coronavirus)[mhe] or ("coronavirus infections")[mhe] | N/A | 42 |
|  | 2 | "SARS-CoV-2" | N/A | 9 |
|  | 3 | coronavirus$ or "corona virus$" | N/A | 4 |
|  | 4 | "Covid-19" or Covid19 | N/A | 37 |
|  | 5 | nCoV | N/A | 0 |
| Remdesivir | 6 | remdesivir or RDV or veklury or GS-5734 or GS5734 | N/A | 2 |
| Combination | 7 | #1 or #2 or #3 or #4 or #5 | N/A | 42 |
|  | 8 | #6 and #7 | N/A | 2 |

**Database:** International HTA to 24th May 2021.

**Abbreviations:** INAHTA: the International Health Technology Assessment database; N/A: not applicable.

**Table 6. Eligibility criteria for CRU studies**

| **Domain** | **Inclusion** | **Exclusion** |
| --- | --- | --- |
| Population | Unselected^a^ adult or adolescent patients (aged ≥12 years) with COVID-19 | - Animal, *in vitro* or *in silico* studies - Selected populations (e.g. pregnant women or patients with cancer) or children <12 years of age - Patients without COVID-19 |
| Intervention | - Remdesivir alone (± SoC) - Remdesivir in combination with other therapies | Any other intervention |
| Comparators | Any comparator (or none) | N/A |
| Outcomes | Both direct and indirect cost/resource use, including but not limited to:  **Direct costs:**   - Hospitalisation costs - Post-discharge costs - Other costs (e.g. cost per COVID-19 test)   **Indirect costs:**   - Productivity losses of patients with COVID-19 - Productivity losses of caregivers for patients with COVID-19 - Productivity losses as a result of government measures due to COVID-19   **Resource use:**   - Hospital length of stay - Number of ICU days - Number of bed days - Treatment use - Ventilatory assistance - Adverse event-related resource use - GP/hospital consultations - Imaging/testing | Studies not presenting relevant outcomes for the population of interest |
| Study design | Any | N/A |
|  | SLRs of relevant primary publications were considered relevant at the title/abstract review stage and hand searched for relevant primary studies, but were excluded during the full-text review stage unless they themselves presented primary research | |
| Publication type | - Original research studies including economic evaluations - HTAs - Congress abstracts published in 2020 | - Any other publication type, including studies not reporting any original research - Congress abstracts published before 2020 |
| Other considerations | - Human subjects - Any language^b^ - Any country | |

**Footnotes**: ^a^Unselected = studies that do not limit to specific populations such as pregnant women or patients with cancer. ^b^Database searches are limited to English sources but any non-English relevant records identified from the searches were included.

**Abbreviations:** CRU: cost and resource use; GP: general practitioner; HTA: health technology assessment; ICU: intensive care unit; N/A: not applicable; SLR: systematic literature review; SoC: standard of care.

**Table 7. Eligibility criteria for economic evaluations**

| **Domain** | **Inclusion** | **Exclusion** |
| --- | --- | --- |
| Population | Unselected^a^ adult or adolescent patients (aged ≥12 years) with COVID-19 | - Animal, *in vitro* or *in silico* studies - Selected populations (e.g. pregnant women or patients with cancer) or children <12 years of age - Patients without COVID-19 |
| Intervention^b^ | - Remdesivir alone (± SoC) - Remdesivir in combination with other therapies | Any other intervention |
| Comparators | Any comparator (or none) | N/A |
| Outcomes | Cost-effectiveness outcomes, including but not limited to:   - ICERs - Cost per clinical outcome - Total QALYs - Total LYGs - Total costs - Incremental costs and QALYs - Cost per death averted | Studies not presenting relevant outcomes for the population of interest |
| Study design | Any of the following analysis types:   - Cost-utility - Cost-effectiveness - Cost-consequence - Cost-benefit - Cost-minimisation | Any other types of analysis |
|  | SLRs of relevant primary publications were considered relevant at the title/abstract review stage and hand searched for relevant primary studies, but were excluded during the full-text review stage unless they themselves presented primary research | |
| Publication type | - Original research studies including economic evaluations - HTAs - Congress abstracts published in 2020 | - Any other publication type, including studies not reporting any original research - Congress abstracts published before 2020 |
| Other considerations | - Human subjects - Any language^c^ - Any country | |

**Footnotes**: ^a^Unselected = studies that do not limit to specific populations such as pregnant women or patients with cancer. ^b^ The search strategies for the original SLR did not include terms for remdesivir as the scope of interventions was originally broader ^c^Database searches were limited to English sources but any non-English relevant records identified from the searches were included.

**Abbreviations:** HTA, health technology assessment; ICER, incremental cost-effectiveness ratio; LYG, life years gained; N/A, not applicable; QALY, quality-adjusted life-year; SLR, systematic literature review; SoC, standard of care.

**Table 8. Quality assessments of CRU studies included in the SLR using the Alberta Heritage Foundation for Medical Research (AHFMR) checklist for quantitative studies**

| **Question** | **Anderson 2021** | **Bechman 2021** | **Béraud 2021** | **Garcia-Vidal 2021** | **Hill 2020** | **ICER 2020** | **Jiang 2021** | **Mozaffari 2021** | **Nasir 2021** | **Sheinson 2021** | **Soriano 2021** |
| --- | --- | --- | --- | --- | --- | --- | --- | --- | --- | --- | --- |
| Was the question/objective sufficiently described? (Yes/Partial/No/NA) | Y – objective clearly described in introduction | Y – objective clearly described in introduction | Y – objective clearly described in introduction | Y – objective clearly described in introduction | Y – objective clearly described in introduction | Y – objective clearly described in introduction | Y – objective clearly described in introduction | Y – objective clearly described in the objective section | Y – objective clearly described in introduction | Y ‒ objective clearly described in introduction | Y – objective clearly described in beginning of the methods |
| Was the study design evident and appropriate? (Yes/Partial/No/NA) | Y – cohort study | Y – observational study | Y – epidemiological model | Y – observational cohort study | Y – study design was a pricing study | Y – study design was a pricing study | Y ‒ cost-effectiveness study | Y – retrospective real-word utilisation study | Y – observational study | Y ‒ cost-effectiveness modelling study | Y – epidemiological model |
| Was the source of information/input variables described and appropriate? (Yes/Partial/No/NA) | Y – participants clearly described and consecutively enrolled | Y – eligibility criteria clearly described | Y – population clearly described and all sources for population data and cost data were given | Y – eligibility criteria clearly described with patients consecutively enrolled | Y – sources for data provided and sources given for the established methods used to estimate minimum manufacturing costs | Partial – sources for data given but unclear how these particular sources were chosen | Y | Y – population clearly described with all patients admitted with ICD-10–CM: U07.1 included | Y – eligibility criteria clearly described | Y ‒ inputs and sources described | Y – population clearly described and all sources for population data were given, and SLR conducted for resource use values |
| Were subject (and comparison group, if applicable) characteristics sufficiently described? (Yes/Partial/No/NA) | Y – baseline demographics reported in table 1 (although not given for the entire cohort these could be easily calculated) | Y – baseline demographic given in table 1 and 2 | N - baseline demographics were not reported | Y – baseline characteristics given for patients (within the month they enrolled – although characteristics for the population as a whole were not presented) | N/A | N/A | N – baseline demographics were not reported | Partially – Only age and sex reported for the different population groups | Partial ‒ some baseline demographics given | N ‒ no details given | N – baseline demographics were not reported |
| If interventional, and random allocation was possible, was it described? (Yes/Partial/No/NA) | N/A | N/A | N/A | N/A | N/A | N/A | N/A | N/A | N/A | N/A | N/A |
| If interventional, and blinding of investigators was possible, was it reported? (Yes/Partial/No/NA) | N/A | N/A | N/A | N/A | N/A | N/A | N/A | N/A | N/A | N/A | N/A |
| If interventional, and blinding of subjects was possible, was it reported? (Yes/Partial/No/NA) | N/A | N/A | N/A | N/A | N/A | N/A | N/A | N/A | N/A | N/A | N/A |
| Were outcome and (if applicable) exposure measure(s) well defined and robust to measurement/misclassification bias? Was the means of assessment reported? (Yes/Partial/No/NA) | Y – definition of LOS was clear | Y – outcomes well defined and robust | Y – outcomes were clearly defined | Y – outcomes were clearly defined | N/A | N/A | N ‒ costs based on assumption | Y – outcomes were clearly defined | Y – outcomes well defined and data collection reported, non-electronic records were used | Partial – outcomes well defined and data collection reported | Y – outcomes were clearly defined |
| Was the sample size appropriate? (Yes/Partial/No/NA) | Y – large sample (1000+) | Y – large sample size (2000+) | Y – country size scale | Y – large sample (1000+) | N/A | N/A | N/A | Y – country size scale | Partial ‒ moderate sample size (99) | Y ‒ for resource use estimates N >1,600 | Y – country size scale |
| Were analytic methods described/justified and appropriate? (Yes/Partial/No/NA) | Y – data abstraction methods referenced, and P values were calculated using the Kruskal-Wallis or Chi-squared tests | Y – analytical methods described in statistical methods section | Y – analytical methods described in statistical methods section | Y –analytical methods described in statistical analysis section | N/A | N/A | N | N – analytical methods not described | Y – analytical methods described in statistical methods section | N – analytical methods not described in statistical methods section | N – analytical methods not described |
| Were estimates of variance reported for the main results? (Yes/Partial/No/NA) | Y – IQR given for median length of stay of entire cohort | Y – p-value reported for number of patients still admitted to hospital | Y – 95% CI reported | Y – IQR given for median time from symptom onset to treatment | N/A | N/A | N | N – no statistical analysis completed | N – no SD or 95% CI, simple n (%) | Y ‒ SE | Y – 95% CI reported |
| Was the study controlled for confounding? (Yes/Partial/No/NA) | N/A | Y – confounding measured controlled for and clearly discussed throughout report | N/A | N/A | N/A | N/A | N | N/A | N | N | N/A |
| Were the results reported in sufficient detail? (Yes/Partial/No/NA) | Y – all results reported clearly | Y – all results reported clearly | Y – results reported and clearly described | Y – results reported and clearly described | Y – results reported and clearly described | Y – results reported and clearly described | Partial – results for the purpose of the review were limited | Y – all results reported clearly | Partial – results for the purpose of the review were limited | Partial – results for the purpose of the review were limited | Y – all results reported clearly |
| Were the conclusions supported by the results? (Yes/Partial/No/NA) | Y | Y | Y | Y | Y | Y – conclusion provided with discussion on results sensitivity | NA ‒ no conclusion related to the cost outcome | Y | Y ‒ conclusions relevant to this review were reported and align with the study results | Partial ‒ conclusions relevant to this review not reported but align with the study results | Y |

**Abbreviations**: CI: confidence interval; CRU: cost and resource use; ICD: International Classification of Diseases; IQR: interquartile range; LOS: length of stay; N: no; N/A: not applicable; SD: standard deviation; SLR: systematic literature review; Y: yes.

**Table 9. Quality assessments of economic evaluations included in the SLR using the Drummond Checklist**

| **Question** | **ICER 2020** | **Jiang 2021** | **Jo 2021** | **Sheinson 2021** |
| --- | --- | --- | --- | --- |
| **Study design** | | | | |
| Was the research question stated? (Yes/No/Unclear/NA) | Y – clearly stated in introduction | Y – clearly stated in introduction | Y – clearly stated in introduction | Y – clearly stated in introduction |
| Was the economic importance of the research question stated? (Yes/No/Unclear/NA) | Y – cost-effectiveness price benchmarks are discussed | Y | Y | Y |
| Was/were the viewpoint(s) of the analysis clearly stated and justified? (Yes/No/Unclear/NA) | Y – healthcare system perspective | Y – healthcare system perspective | Y – healthcare system perspective | Y ‒ multiple perspectives |
| Was a rationale reported for the choice of the alternative programs or interventions compared? (Yes/No/Unclear/NA) | Y – standard of care used and updated based on WHO guidance | Y – standard of care based on treatment of COVID in China | Y – adding dexamethasone and remdesivir relative to the standard care | N ‒ limited information on the interventions (remdesivir and dexamethasone) in the treated group |
| Were the alternatives being compared clearly described? (Yes/No/Unclear/NA) | Y – remdesivir clearly described and dexamethasone described in appendix table 1 | Partly – specific interventions used as SoC in China described based on published studies, however dosages and regimens were not described for remdesivir or SoC | N – standard care not specified/described | N – standard care not specified/described |
| Was the form of economic evaluation stated? (Yes/No/Unclear/NA) | Y – cost-effectiveness | Y – cost-effectiveness | Y – cost-effectiveness | Y ‒ cost-effectiveness |
| Was the choice of form of economic evaluation justified in relation to the questions addressed? (Yes/No/Unclear/NA) | Y – to estimate the lifetime costs and outcomes of remdesivir | Y – to estimate the costs and QALYs of remdesivir | Y – to estimate costs and deaths averted | Y – to estimate the costs and QALYs of potential treatment |
| **Data collection** | | | | |
| Was/were the source(s) of effectiveness estimates used stated? (Yes/No/Unclear/NA) | Y – sources given in text and appendix table 1 | Y – inputs and sources reported | Y ‒ inputs and sources reported | Y ‒ inputs and sources reported |
| Were details of the design and results of the effectiveness study given (if based on a single study)? (Yes/No/Unclear/NA) | Partly – results given but study designs were not reported in any detail | N – details and results not given, only referenced | N – details and results not given, only referenced | N – details and results not given, only referenced |
| Were details of the methods of synthesis or meta-analysis of estimates given (if based on an overview of a number of effectiveness studies)? (Yes/No/Unclear/NA) | N – methods for choosing sources were not reported | N – an OR for clinical improvement for a meta-analysis was provided and cited but no further details | N/A ‒ no meta-analysis reported | N/A ‒ no meta-analysis reported |
| Were the primary outcome measure(s) for the economic evaluation clearly stated? (Yes/No/Unclear/NA) | Y – ICER per QALY | Y – ICER per QALY | Y ‒ ICER per deaths averted | Y – ICER per QALY |
| Were the methods used to value health states and other benefits stated? (Yes/No/Unclear/NA) | N – utility values given along with sources but details on methods used to value health states was not | N – utility values given along with sources but details on methods used to value health states was not | N/A ‒ clinical/mortality only | Y ‒ scores were mapped to EQ-5D utilities using the Ara and Brazier algorithm |
| Were the details of the subjects from whom valuations were obtained given? (Yes/No/Unclear/NA) | N – only sources provided | N – only sources provided | N – only sources provided | N – only sources provided |
| Were productivity changes (if included) reported separately? (Yes/No/Unclear/NA) | N/A | N/A | N/A | Y |
| Was the relevance of productivity changes to the study question discussed? (Yes/No/Unclear/NA) | N/A | N/A | N/A | Y ‒ productivity changes discussed |
| Were quantities of resources reported separately from their unit cost? (Yes/No/Unclear/NA) | Y – costs reported alongside resource use (percent requiring types of oxygen support and duration of hospitalisation reported in appendix table 1) | Partly – costs are reported in Table 2 | Y – costs and healthcare utilisation reported | Y ‒ reported in Table 2 |
| Were the methods for the estimation of quantities and unit costs described? (Yes/No/Unclear/NA) | Y – sources for cost data reported | Y – sources for cost data reported | Y – sources for cost data reported | Y |
| Were currency and price data recorded? (Yes/No/Unclear/NA) | Partly – currency given but cost year was not reported | Y – currency and cost year reported | Y – currency and cost year reported | Y ‒ currency and cost year reported |
| Were details of price adjustments for inflation or currency conversion given? (Yes/No/Unclear/NA) | N – no details on conversion or inflation given | N – no details on conversion or inflation given | Y – details on conversion given | Y ‒ inflated using the medical care services component of the consumer price index, USD |
| Were details of any model used given? (Yes/No/Unclear/NA) | Y – Markov model | Y – dynamic compartment transmission model | N ‒ no/little information on model structure | Y ‒ decision tree and Markov |
| Was there a justification for the choice of model used and the key parameters on which it was based? (Yes/No/Unclear/NA) | N – no discussion was had on type of model used | Y – the structure of the model was discussed and justified | N | Y – the structure of the model was discussed and justified |
| **Analysis and interpretation of results** | | | | |
| Was the time horizon of cost and benefits stated? (Yes/No/Unclear/NA) | Y – lifetime | Y – time horizon reported as 55-days (with additional calculations for QALY gain due to decreased mortality) | N – time horizon unclear from the article, although appears to be from August 2020 to January 2021 | Y ‒ lifetime |
| Was the discount rate stated? (Yes/No/Unclear/NA) | Y – 3% | Y – 5% | Y – 5% | Y ‒ all costs and health effects discounted at 3% per year |
| Was the choice of rate justified? (Yes/No/Unclear/NA) | N | Y – as per the China Guideline for Pharmacoeconomic Evaluation | Y – as per South African pharmacoeconomic guidelines | Partly – referenced a report on recommendations on CEA |
| Was an explanation given if cost or benefits were not discounted? (Yes/No/Unclear/NA) | N/A | N/A | N/A | N/A |
| Were the details of statistical test(s) and confidence intervals given for stochastic data? (Yes/No/Unclear/NA) | N – no CI or SDs reported | N – no CI or SDs reported | Y ‒ 95% CIs | Y ‒ 95% CIs reported |
| Was the approach to sensitivity analysis described? (Yes/No/Unclear/NA) | Y – scenario analysis | Y – one-way sensitivity and probabilistic sensitivity analyses | Y – one- and three-way sensitivity analyses and probabilistic sensitivity analysis | Y ‒ one-way and probabilistic sensitivity analyses |
| Was the choice of variables for sensitivity analysis justified? (Yes/No/Unclear/NA) | Y – it is discussed in the report why the two supporting scenarios were conducted | Y – variables discussed and justified | Y ‒ all parameters | Y |
| Were the ranges over which the parameters were varied stated? (Yes/No/Unclear/NA) | Y – assumption of scenarios clearly discussed | Y – assumption of scenarios clearly discussed | Y – assumption of scenarios clearly discussed | N ‒ no justification |
| Were relevant alternatives compared in the incremental analysis? (Yes/No/Unclear/NA) | Y – remdesivir vs standard of care | Y – remdesivir vs standard of care | Y – remdesivir vs standard of care | Y ‒ treatment vs standard of care |
| Was an incremental analysis reported? (Yes/No/Unclear/NA) | Y – ICER per QALY | Y – ICER per QALY | Y – ICER per death averted | Y – ICER per QALY |
| Were major outcomes presented in a disaggregated as well as aggregated form? (Yes/No/Unclear/NA) | Y – ICER reported alongside total costs, QALYs and LYG | Y – ICER reported alongside total costs, QALYs | Y – ICER reported alongside total costs, deaths averted | Y – ICER reported alongside total costs, QALYs |
| Was the answer to the study question given? (Yes/No/Unclear/NA) | Y – remdesivir cost needed to be cost-effective is reported | Y – remdesivir cost needed to be cost-effective is reported | Y – remdesivir cost-effectiveness | Y – treatment cost-effectiveness compared to BSC |
| Did conclusions follow from the data reported? (Yes/No/Unclear/NA) | Y | Y – the ICER was below the WTP threshold | Y | Y |
| Were conclusions accompanied by the appropriate caveats? (Yes/No/Unclear/NA) | Y – limitations discussed | Y – limitations discussed | Y ‒ limitations discussed | Y – limitations discussed |

**Abbreviations:** BSC: best supportive care; CEA: cost-effectiveness analysis; CI: confidence interval; ICER: incremental cost-effectiveness ratio; ICER: Institute for Clinical and Economic Review; LYG: life-year gain; N: no; N/A: not applicable; OR: odds ratio; QALY: quality-adjusted life year; SD: standard deviation; SoC: standard of care; USD United States dollar; WHO: World Health Organisation; WTP: willingness-to-pay; Y: yes.
